# Restoration pathways reshape forest structure and multifunctionality in subtropical plantations and secondary forests

**DOI:** 10.3389/fpls.2026.1941032

**Published:** 2026-10-21

**Authors:** Juyang Wu, Qin Xiang, Xuemei Wang, Shan Zhou, Jingpin Lei

**Affiliations:** 1 Key Laboratory of Forest Cultivation and Management of National Forestry and Grassland Administration, Research Institute of Forestry, Chinese Academy of Forestry, Beijing, China; 2 Faculty of Resources and Environmental Science, Hubei University, Wuhan, China; 3 College of Forestry, Southwest Forestry University, Kunming, China

**Keywords:** *Cunninghamia lanceolata*, ecosystem multifunctionality, ecosystem service imbalance, restoration pathway, service bundle, stand structure

## Abstract

Forest restoration may enhance ecosystem multifunctionality (EMF) without improving all ecosystem services equally. We compared 36 plots in 32–39-year-old stands across four subtropical regions of southern China, representing pure *Cunninghamia lanceolata* plantations, *C. lanceolata*–broadleaved mixed plantations, and naturally regenerated secondary forests following plantation clear-cutting. Eight service indicators represented soil conservation, water conservation, canopy-based climate regulation, carbon storage, stand productivity, tree species diversity, regeneration capacity, and soil fertility maintenance. EMF was quantified using averaging and threshold-based approaches, while root mean square deviation measured pairwise service imbalance. Regression models, relative-importance analysis, and structural equation modeling were used to examine associations between environmental and stand attributes and EMF. Mixed plantations had the highest mean EMF (0.559), compared with secondary forests (0.403) and pure plantations (0.383), and significantly higher multifunctionality at the 50% and 75% thresholds. However, they also had the largest number of highly imbalanced service pairs. Differences at the 25% threshold were not significant. Regeneration capacity was relatively low across all pathways, whereas static water retention was higher in secondary forests than in either plantation type. Prominent correlates differed among pathways: soil total phosphorus and species mingling in pure plantations, diameter-based structural diversity and stand age in mixed plantations, and stand density and soil total nitrogen in secondary forests. Higher aggregate EMF therefore did not imply a more balanced service bundle. This observational comparison supports evaluating restoration through both aggregate multifunctionality and individual service profiles, with inference restricted to the sampled middle-aged stands.

## 1 Introduction

Forests are a major component of the terrestrial biosphere, covering approximately 31% of the global land surface and supporting high biodiversity, complex stand structures, and multiple ecological processes (Bonan, 2008). Under global change, forests are increasingly expected to provide multiple ecosystem services simultaneously, including carbon sequestration, water regulation, soil conservation, biodiversity maintenance, nutrient cycling, timber production, and natural regeneration (Costa et al., 2026; Nabhani et al., 2024). The shift from production-oriented to multifunctional forest management requires assessing both individual ecosystem services and the capacity of forests to sustain multiple functions simultaneously. This capacity is commonly conceptualized as forest ecosystem multifunctionality (EMF) (Manning et al., 2018). However, multifunctionality does not imply the synchronous enhancement of all ecosystem services. Biodiversity–ecosystem functioning research has shown that forests with higher tree species richness often maintain higher levels of multiple ecosystem services, yet no single tree species, functional group, or stand attribute can optimize all services simultaneously (Hua et al., 2022; Perez-Verdin et al., 2018). In species-rich subtropical forests, EMF also depends on multiple dimensions of biodiversity, functional trait composition, and trophic interactions; tree species richness alone therefore cannot fully explain variation in multifunctionality (Beugnon et al., 2025; Schuldt et al., 2018). Therefore, evaluating forest management outcomes requires an explicit understanding of how ecosystem services are bundled, coordinated, and traded off during forest restoration (Bennett et al., 2009; Byrnes et al., 2014).

*Cunninghamia lanceolata* is among the most extensively planted and commercially important timber species in China (Brown, 2000; Farooq et al., 2020); its plantations have been estimated to account for approximately 24% of China’s plantation area and 6.1% of the global plantation area (Li et al., 2021). *C. lanceolata* plantations contribute substantially to industrial timber supply, afforestation, and regional carbon sequestration. However, long-term monoculture management and successive rotations are frequently associated with declining productivity, limited natural regeneration, soil nutrient depletion, and degradation of soil microbial community structure and metabolic activity (Fang et al., 2026; Wu et al., 2017; Zhang et al., 2025a). To mitigate these constraints, restoration of *C. lanceolata* plantations has increasingly emphasized the conversion of single-species coniferous plantations into conifer–broadleaved mixed stands, as well as the promotion of natural recovery toward secondary forests. These restoration pathways are expected to enhance stand structural complexity, resource-use complementarity, and belowground biogeochemical processes (Li et al., 2022). Nevertheless, plantation conversion does not necessarily coincide with the synchronous enhancement of all ecosystem services. Recent studies have shown that mixed plantations do not always exhibit higher EMF than monocultures, whereas secondary forests may outperform managed plantations in specific functional dimensions, such as nutrient cycling, carbon storage, water regulation, and decomposition processes (Li et al., 2024a, b; Zhou et al., 2024). Therefore, the ecological consequences of *C. lanceolata* plantation restoration should not be evaluated solely according to whether mixed plantations or secondary forests exceed pure plantations in overall multifunctionality. Greater attention should be given to how ecosystem-service bundles and functional bottlenecks differ among restoration pathways.

The formation of ecosystem service bundles and EMF during plantation restoration is likely governed by the coordination, or mismatch, among stand structure, species composition, soil conditions, and resource competition. Stand structural attributes regulate the spatial distribution of light, water, nutrients, and growing space, thereby influencing aboveground productivity, carbon storage, regeneration dynamics, and hydrological processes (Liang et al., 2016). Taxonomic and functional diversity may enhance EMF through niche complementarity, facilitation, and more complete resource acquisition, but these effects are often contingent on stand structural configuration and environmental constraints (Li et al., 2021; Ouyang et al., 2023). Soil nutrient availability provides the site-level foundation for biomass accumulation, soil fertility maintenance, and microbially mediated nutrient cycling (Zhang et al., 2026), whereas climatic water deficit may suppress tree growth, water conservation, and soil biogeochemical activity by intensifying water limitation (Zhang et al., 2025b). Importantly, the same structural or compositional change may generate both service complementarity and service conflict. For example, greater stand complexity may enhance carbon storage, biodiversity, and microclimate buffering, while the associated changes in resource availability may affect tree growth, natural regeneration, and water regulation differently. Evaluating EMF therefore requires examining whether its associations with soil, diversity, and stand attributes vary across low, intermediate, and high multifunctionality thresholds (Byrnes et al., 2014).

Despite substantial progress in forest multifunctionality research, several knowledge gaps remain in plantation restoration systems. First, many assessments of forest restoration still focus primarily on individual ecosystem services or aggregate EMF indices, whereas insufficient attention has been paid to how ecosystem services form bundles and which services constitute relative functional bottlenecks (Zhang et al., 2025c). Second, higher aggregate EMF does not necessarily indicate greater coordination among component services, because it may arise from improvements in only a subset of services while other functions remain constrained. It therefore remains uncertain whether any EMF advantage of mixed plantations is accompanied by weaker service imbalances (Felipe-Lucia et al., 2018). Third, although stand structure, species composition, spatial heterogeneity, soil conditions, and plant diversity are associated with the supply of multiple ecosystem services, their relationships with EMF may differ among restoration pathways and multifunctionality thresholds (Chen et al., 2025; Costa et al., 2026). These gaps limit our ability to identify whether restoration improves multifunctionality broadly, redistributes performance among services, or leaves particular functions constrained. They are especially relevant to subtropical *C. lanceolata* plantation landscapes, where restoration is expected to reconcile timber production, carbon sequestration, soil conservation, hydrological regulation, soil fertility maintenance, biodiversity conservation, and natural regeneration.

To address these knowledge gaps, this study used an observational design to compare 32–39-year-old stands representing three restoration pathways in subtropical China: pure *C. lanceolata* plantations (PF), *C. lanceolata*–broadleaved mixed plantations (MF) restored through closer-to-nature management, and naturally regenerated secondary forests (NF) following clear-cutting of *C. lanceolata* plantations. We quantified eight ecosystem services using indicators representing provisioning, regulating, and supporting dimensions. Root mean square deviation (RMSD) was used to evaluate pairwise service imbalances rather than statistical trade-offs or synergies. Aggregate EMF was quantified using averaging and threshold-based approaches. Specifically, we tested four hypotheses. H1: ecosystem-service bundle configurations differ among restoration pathways. H2: mixed plantations have higher aggregate EMF than pure plantations and secondary forests, but do not necessarily exhibit lower pairwise service imbalance. H3: regeneration capacity and water conservation are recurrent relative functional bottlenecks, although their severity differs among pathways. H4: the attributes associated with EMF differ among restoration pathways and multifunctionality thresholds.

## 2 Materials and methods

### 2.1 Study area and sample plots

This observational study was conducted in four subtropical regions of southern China: Enshi, Hubei Province (29°30′–31°24′ N, 108°23′–110°38′ E); Huitong, Hunan Province (26°40′–27°09′ N, 109°26′–109°47′ E); Longnan, Jiangxi Province (24°29′–25°07′ N, 114°47′–115°34′ E); and Pingxiang, Guangxi Zhuang Autonomous Region (22°02′–22°37′ N, 106°41′–106°59′ E) (**Figure 1**). These regions have a warm, humid subtropical monsoon climate, with mean annual temperatures of approximately 16–22 °C and annual precipitation generally ranging from 1200 to 1700 mm. Precipitation occurs mainly from April to September. The terrain is dominated by mountains and hills, and the prevailing soil types are red and yellow soils derived primarily from weathered granite or sandstone parent materials. The zonal vegetation consists mainly of subtropical evergreen broadleaved forests or evergreen–deciduous broadleaved mixed forests. Extensive afforestation for timber production has made *C. lanceolata* plantations a major component of these forest landscapes.

**Figure 1:**
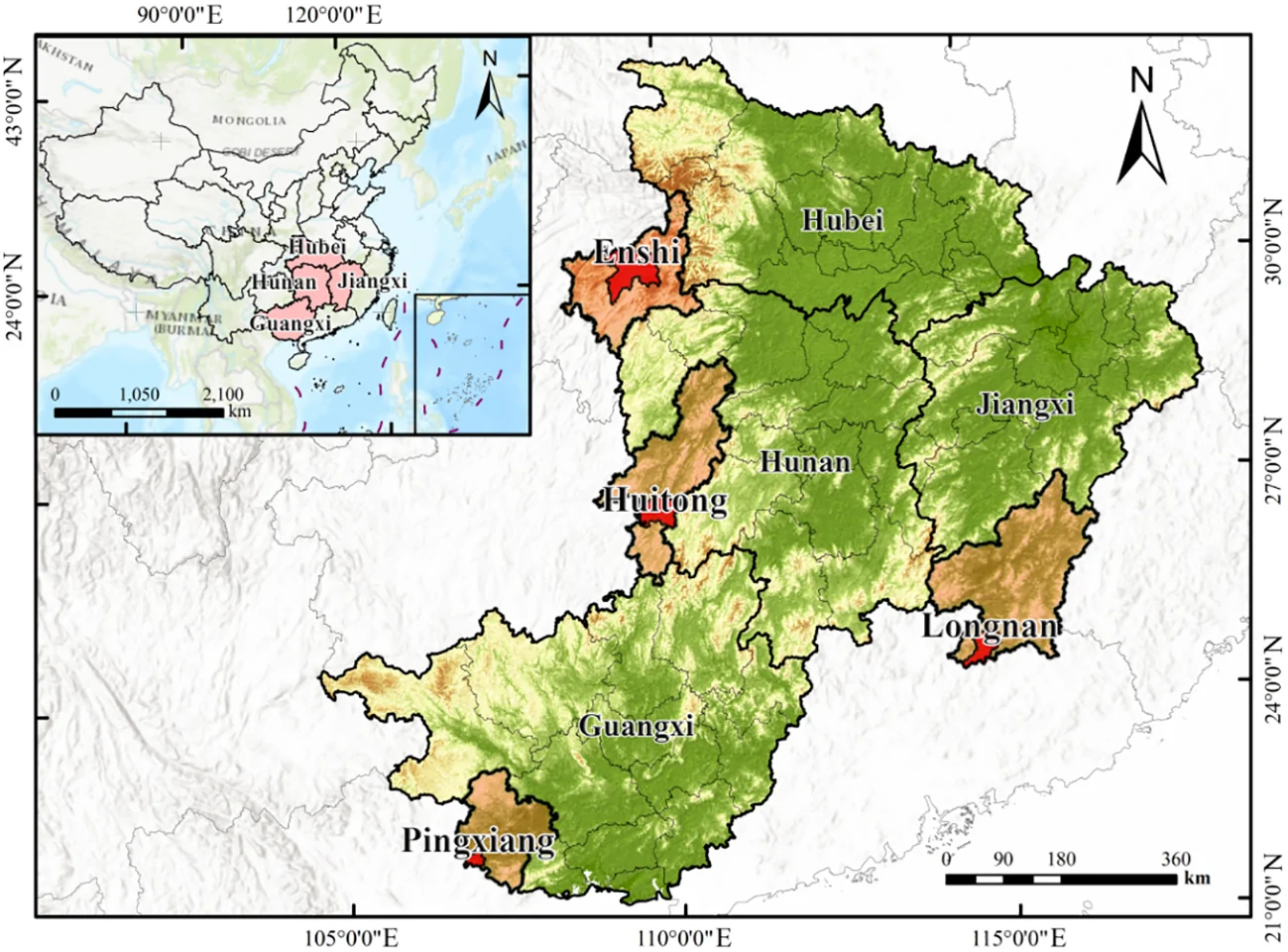
Location of the study areas in subtropical China.

From July to September 2025, stands representing three restoration pathways were selected in each region: pure *C. lanceolata* plantations (PF), which were artificially established stands dominated by *C. lanceolata*; *C. lanceolata*–broadleaved mixed plantations (MF), developed through thinning of planted *C. lanceolata* stands followed by broadleaved enrichment planting; and naturally regenerated secondary forests (NF), established through natural succession after clear-cutting of former *C. lanceolata* plantations. No further tending or other silvicultural treatments were applied after these pathway-defining interventions. Detailed information on stand origin, regeneration source, and silvicultural history is provided in **Supplementary Table S2**.

Stand selection was based on historical management records, expert consultation, and field reconnaissance to identify stands representative of each restoration pathway in each region. Stand age was determined from records of plantation establishment or recovery initiation and cross-validated against the mean age of sampled canopy trees. The sampled stands were 32–39 years old, with a mean age of 35.0 years and a standard deviation (SD) of 2.6 years. Restricting stand selection to this relatively narrow age range improved developmental comparability among restoration pathways, although residual age-related variation could not be completely eliminated. Inference was therefore restricted to the sampled middle-aged stands.

For each restoration pathway, three 20 m × 20 m permanent plots were established in each region, yielding a total of 36 plots. All plots were separated by at least 100 m to minimize spatial dependence among plots. Each 20 m × 20 m plot was divided into four 10 m × 10 m subplots, and each subplot was further divided into four 5 m × 5 m quadrats to facilitate tree-level georeferencing, vegetation surveys, and fine-scale analysis of stand spatial structure.

Plot-level geographic and site information is provided in **Supplementary Table S1**, whereas stand characteristics and management histories summarized by region and restoration pathway are provided in **Supplementary Table S2**. Detailed census protocols and measured variables are provided in Supporting Information S1.

### 2.2 Selection of biotic and abiotic variables

To examine the environmental and stand attributes associated with ecosystem service bundles and ecosystem multifunctionality (EMF) across restoration pathways, we compiled a set of candidate biotic and abiotic variables representing regional climate, soil conditions, stand structure, spatial configuration, and tree species diversity. These variables describe environmental conditions and stand attributes potentially associated with resource availability, competition, nutrient cycling, and forest regeneration.

The initial variable set included 24 indicators covering climatic factors, soil properties, stand attributes, and diversity-related metrics (Supporting Information S1.3). To reduce multicollinearity, pairwise Pearson correlations were first calculated among all candidate variables. When two variables were strongly correlated (|*r*| > 0.70), one was retained according to its ecological relevance, interpretability, and expected association with EMF (Yuan et al., 2021). Variance inflation factors (VIFs) were subsequently calculated for the reduced variable set, and only variables with VIF values < 3.5 were retained for subsequent analyses. Based on this screening procedure and previous ecological evidence (Costa et al., 2026; Lan et al., 2023; Sanaei et al., 2021; Wu et al., 2025), ten variables were ultimately retained: mean annual temperature (MAT), soil pH (pH), soil total nitrogen (STN), soil total phosphorus (STP), mean stand age (Age), stand density index (SDI), mingling degree (M_m_), Hegyi competition index (CI_m_), tree species richness (SR), and diameter-based structural diversity (SDN).

These variables were treated as candidate explanatory covariates rather than experimentally verified causal drivers. Model estimates were therefore interpreted as statistical associations that could inform ecological explanations, without establishing the underlying mechanisms.

### 2.3 Quantification of ecosystem services and multifunctionality

Following the Millennium Ecosystem Assessment (2005) framework, we selected eight ecosystem services (ESs) representing provisioning, regulating, and supporting dimensions relevant to the restoration of *C. lanceolata* plantations. Cultural services were not included because the plot-level biophysical measurements used in this study did not directly quantify stakeholder demand, recreational use, or other non-material benefits. The selected ESs included soil conservation (SC), water conservation (WC), canopy-based climate regulation (CR), carbon storage (CS), stand productivity (SP), tree species diversity (H′), regeneration capacity (RC), and soil fertility maintenance (SF) (**Table 1**).

**Table 1:** Ecosystem services, ecological significance, and quantitative indicators used in this study.

Service category	Ecosystem service	Abbreviation	Ecological significance and scope of interpretation	Quantitative indicator
Regulating	Soil conservation	SC	Reflects soil retention that helps maintain rooting conditions and site productivity, assessed through modeled reductions in soil erosion.	Difference between potential and actual annual soil erosion (t·ha^-1^·yr^-1^)
	Water conservation	WC	Represents litter–soil water-storage potential relevant to hydrological buffering, assessed as static capacity rather than realized runoff regulation or groundwater recharge.	Combined maximum water-holding capacity of litter and sampled 0–10 cm mineral soil (t·ha^-1^)
	Canopy-based climate regulation	CR	Reflects canopy shading and radiation-interception potential relevant to understory microclimate buffering. Temperature and humidity were not measured directly.	Mean of min–max-normalized leaf area index and canopy projected cover
	Carbon storage	CS	Quantifies carbon retained in the measured vegetation, litter, and surface-soil pools at the survey date.	Combined vegetation, litter, and 0–20 cm mineral-soil carbon stocks (t·ha^-1^)
Provisioning	Stand productivity	SP	Provides an age-averaged standing-volume proxy for timber-production potential, based on the current standing stock.	Standing volume divided by stand age (m^3^·ha^-1^·yr^-1^)
Supporting	Tree species diversity	H′	Captures tree species richness and relative dominance, representing the taxonomic diversity of the surveyed tree layer.	Shannon–Wiener index weighted by species proportions of plot basal area
	Regeneration capacity	RC	Reflects the established natural regeneration pool available for subsequent stand development, assessed at the survey date.	Density of naturally regenerated tree saplings with *DBH*< 4 cm, censused throughout each 20 × 20 m plot (stems ha^-1^)
	Soil fertility maintenance	SF	Represents the organic-matter foundation of fertility maintenance through its links to nutrient retention, soil structure, and microbial processes. Nutrient-specific availability and cation exchange capacity are outside the indicator’s scope.	Soil organic carbon stock in the 0–20 cm mineral-soil layer (t·ha^-1^)

*DBH*, diameter at breast height. The indicators quantify the biophysical dimensions specified above. Their calculation methods and interpretation are described in Sections 2.3.1–2.3.8.

Together, these indicators encompass widely assessed forest services, including stand productivity and carbon storage, as well as restoration-sensitive functions, such as water conservation, soil fertility maintenance, and regeneration capacity. They represent selected biophysical dimensions of the corresponding services rather than comprehensive measures of service provision. A supplementary correlation analysis characterized covariation among the eight ES indicators without changing their inclusion or weighting in EMF (Section S1.5; **Supplementary Figure S6**). All indicators were oriented so that higher values consistently represented higher performance in the corresponding service dimension before normalization. The abbreviations and quantitative indicators used for the eight services are summarized in **Table 1**.

#### 2.3.1 Soil conservation

Soil conservation (SC) is an important regulating service of forest ecosystems and represents the capacity of vegetation, litter, and soil surface properties to reduce soil loss caused by rainfall-induced erosion (Ausseil et al., 2013). In this study, SC was quantified as the difference between potential and actual soil erosion (Mirakhorlo and Rahimzadegan, 2020).

Potential and actual soil erosion were estimated using the Universal Soil Loss Equation (USLE), which integrates rainfall erosivity, soil erodibility, slope length, slope steepness, vegetation cover, and conservation-practice factors (Ozsoy et al., 2012; Sonneveld and Nearing, 2003). Rainfall erosivity was calculated using the monthly precipitation method proposed by Wischmeier and Smith (1958), and soil erodibility was estimated using the Erosion–Productivity Impact Calculator (EPIC) model. Because no specific engineering soil-conservation measures were implemented in the study plots, the conservation practice factor was set to 1 (Wang and Dai, 2020). The vegetation cover factor was approximated using leaf area index (LAI). The difference between potential and actual erosion provided a model-based indicator of soil retention under the observed site and vegetation conditions. The equations and parameter definitions are provided in Equations 1-1 to 1-9.

(1-1) $$SC=SC_{P}-SC_{A}$$

(1-2) $$SC_{A}=R\times K\times L\times S\times C\times P$$

(1-3) $$SC_{P}=R\times K\times L\times S$$

where SC is the amount of soil conserved (t·ha^-1^·yr^-1^), *SC_A_* is the actual soil erosion per unit area (t·ha^-1^·yr^-1^), *SC_P_* is the potential soil erosion per unit area (t·ha^-1^·yr^-1^), *R* is the rainfall erosivity factor (MJ·mm·ha^-1^·ha^-1^·yr^-1^), *K* is the soil erodibility factor, *L* is the slope length factor, *S* is the slope steepness factor, *C* is the vegetation cover factor, and *P* is the conservation practice factor.

(1-4) $$R=\sum_{i=1}^{12}\left[1.735\times 10^{1.51g\frac{ji^{2}}{j}-0.8188}\right]$$

where *J* denotes annual precipitation, and *j_i_* denotes monthly precipitation (*i* = 1, 2, 3,…, 12).

(1-5) $$\begin{array}{l} K=\left[0.2+0.3\times e^{-0.256ms(1-\frac{msilt}{100})}\right]\times {\left(\frac{msilt}{mc+msilt}\right)}^{0.3}\times \frac{1-0.25_{org}}{orgC+e^{3.72-2.95_{org}C}}\\ \times \left[\frac{0.7\times (1-ms/100)}{(1-ms/100)+e^{-5.51+22.9(2-m_{s}/100)}}\right] \end{array}$$

where *m_c_*, *m_silt_*, *m_s_*, and *_org_C* represent the clay fraction (< 0.002 mm), silt fraction (0.002–0.05 mm), sand fraction (> 0.05 mm), and organic carbon content (%), respectively.

(1-6) $$C={\text{e}}^{\text{ai}\cdot LAI}$$

where *C* is the dimensionless vegetation cover factor, LAI is leaf area index, and a_i_ is a vegetation-specific coefficient set to −0.1535 for forestland (Zhang et al., 2018).

(1-7) $$L={\left(\frac{\lambda }{22.13}\right)}^{\beta /(1+\beta )}$$

(1-8) $$\beta =\frac{\sin\theta /0.089}{3.0\times {\left(\sin\theta \right)}^{0.8}+0.56}$$

(1-9) $$S=\left\{\begin{array}{l} 10\sin\theta +0.03\ldots \ldots .\left(\theta <5.14{}^{\circ}\right)\\ 16.8\sin\theta -0.5\ldots \ldots .\left(5.14{}^{\circ}\leq \theta <10.20{}^{\circ}\right)\\ 21.91\sin\theta -0.96\ldots \left(10.20{}^{\circ}\leq \theta <28.81{}^{\circ}\right)\\ 9.5988\ldots \ldots \ldots \ldots \ldots \ldots .\left(\theta \geq 28.81{}^{\circ}\right) \end{array}\right\}$$

where m is the slope length exponent, *λ* is the slope length (m), and *θ* is the slope angle (°).

#### 2.3.2 Water conservation

Water conservation (WC) represents the capacity of forest litter and soil to retain water, regulate runoff, and buffer hydrological variability (Rolo et al., 2021). In forest stands, the litter layer can delay surface runoff and reduce soil evaporation, whereas the soil layer serves as the primary water-storage reservoir by retaining water and facilitating infiltration (Zhu and Cheng, 2022). We assessed the water-retention component of this service using the combined maximum water-holding capacity of the litter layer and the 0–10 cm mineral-soil layer, expressed in t·ha^-^¹. This indicator characterizes the potential storage capacity of these two reservoirs and provides a common basis for comparing static water retention among restoration pathways. Runoff generation, runoff regulation, and groundwater recharge were outside the scope of this assessment.

#### 2.3.3 Canopy-based climate regulation

Canopy-based climate regulation (CR) represents the potential of forest canopies to buffer understory microclimatic variation through shading and radiation interception. Leaf area index (LAI) and canopy projected cover were used as complementary structural indicators of this potential. LAI characterizes the leaf-area basis for radiation interception and vegetation–atmosphere exchange, whereas canopy projected cover represents stand-level shading potential. Because understory temperature and humidity were not measured directly, CR was interpreted as canopy-based microclimate-buffering potential. For each plot, canopy projected cover was calculated from individual tree crown width and plot area. LAI and canopy projected cover were then separately rescaled to the 0–1 range, and their mean value was used as the CR indicator. The equations and parameter definitions are provided in Equations 2-1 and 2-2.

(2-1) $$CR=\frac{LAI_{\text{i}}^{\text{std}}+CC_{\text{i}}^{\text{std}}}{2}$$

(2-2) $$CC\text{i}=\min(1,\frac{\sum_{k=1}^{n}\pi {\left(\frac{CWik}{2}\right)}^{2}}{A})$$

where *CW_ik_* denotes the mean crown width of tree *k* in plot *i*, *A* is plot area (m^2^), *n* is the number of trees in the plot, and *CC_i_* is canopy projected cover. The superscript “std” denotes min–max normalization to the 0–1 range.

#### 2.3.4 Carbon storage

Carbon storage (CS) was quantified as the total carbon stock in vegetation, litter, and the 0–20 cm mineral-soil layer, representing carbon retained in these pools at the survey date. Soil carbon stock was calculated from soil organic carbon concentration, bulk density, and gravel fraction for the 0–20 cm mineral-soil layer, together with the corresponding soil depth. Litter carbon stock was calculated from litter standing dry mass and carbon concentration. Vegetation carbon stock comprised tree, shrub, and herbaceous components. Tree and shrub biomass was estimated using species-specific allometric equations for stems, branches, foliage, and roots based primarily on diameter at breast height (*DBH*). Herbaceous biomass was determined by destructive harvesting, oven-drying the samples at 80 °C to constant mass, and weighing the resulting dry material. Biomass was converted to carbon stock using species- and organ-specific carbon concentration coefficients obtained from national forestry standards and published sources (Li and Lei, 2010). The calculation procedures are summarized in Equations 3-1 to 3-4.

(3-1) $$CS=CS_{\text{s}}+CS_{\text{d}}+{\text{CS}}_{\text{p}}$$

(3-2) $$CS_{\text{s}}=C_{\text{si}}\times B_{\text{i}}\times H_{\text{i}}\times \left(1-G_{\text{i}}\right)$$

(3-3) $$CS_{\text{d}}=C_{di}\times S_{di}$$

(3-4) $$CS_{\text{p}}=B{\text{t}}_{\text{i}}\times C_{\text{si}}+B{\text{sh}}_{\text{i}}\times C_{\text{si}}+B{\text{h}}_{\text{i}}\times C_{\text{si}}$$

where *CS_s_*, *CS_d_*, and *CS_p_* represent carbon stocks in the soil, litter, and vegetation layers, respectively. *C_si_* denotes soil carbon content (%), *B_i_* is soil bulk density (g·cm^-3^), *H_i_* is soil depth (cm), and *G_i_* is the gravel fraction (%); carbon stocks are expressed in t·ha^-1^. For the litter layer, carbon stock was calculated from litter carbon content (%) and litter standing biomass (*S_di_*, t·ha^-1^). Total vegetation biomass was estimated as the sum of tree biomass (*B_ti_*), shrub biomass (*B_shi_*), and herbaceous biomass (*B_hi_*).

#### 2.3.5 Stand productivity (SP)

Stand productivity (SP) was represented by standing volume divided by stand age, an annualized proxy for timber-production potential expressed in m^3^·ha^-1^·yr^-1^, and was calculated using Equation 4-1:

(4-1) $$SP=\frac{V}{A\text{ge}}$$

where *V* is standing volume per unit area (m³·ha^-1^) and Age is stand age (years).

#### 2.3.6 Species diversity

Tree species diversity was quantified using the Shannon–Wiener diversity index (H′). The relative abundance of each tree species was calculated as its proportion of total basal area within each plot. Basal-area-weighted H′ captures tree species richness and differences in species dominance within the surveyed tree layer. This index differs from tree species richness (SR), which was included as a candidate stand covariate in Section 2.2 and represents only the number of tree species present. H′ was calculated using Equation 5-1:

(5-1) $$H'=-\sum_{i=1}^{n}p_{i}\text{ln}p_{i}$$

where *n* is the number of tree species in a plot and *p_i_* is the proportion of total basal area contributed by species *i*.

#### 2.3.7 Regeneration capacity

Natural regeneration was included as a supporting dimension of forest multifunctionality (Jönsson and Snäll, 2020). In this study, regeneration capacity (RC) was quantified as the density of naturally regenerated tree saplings with *DBH* < 4 cm censused throughout each 20 × 20 m plot. Established saplings constitute a regeneration pool available for subsequent stand development, and their abundance is an informative measure of regeneration status (Miller et al., 2023). Sapling density was converted to stems·ha^-1^ before analysis. Higher RC values therefore indicate a greater abundance of established natural regeneration at the survey date, providing a field-based measure of the current regeneration pool across restoration pathways.

#### 2.3.8 Soil fertility maintenance

Soil fertility maintenance (SF) was represented by soil organic carbon (SOC) stock in the 0–20 cm mineral-soil layer, used as a proxy for the organic-matter foundation of soil fertility. Soil organic matter contributes to nutrient retention, aggregate stability, and microbial processes involved in nutrient cycling, providing an ecological basis for this indicator (Lal, 2016). SOC stock was therefore used to compare the organic-matter-related component of fertility maintenance among restoration pathways. Nutrient-specific availability, including nitrogen, phosphorus, and potassium, and cation exchange capacity were not incorporated into the SF indicator. SF was calculated using soil organic carbon concentration, bulk density, sampling depth, and gravel fraction (Equation 6-1):

(6-1) $$SF=\frac{1}{10}\sum_{i=1}^{n}SOC_{i}\times B_{i}\times H_{i}\times (1-G_{i})$$

where *SOC_i_* denotes the soil organic carbon content of plot *i* (g·kg^-1^), *B_i_* represents soil bulk density (g·cm^-3^), *H_i_* is the soil sampling depth (cm), and *G_i_* is the gravel fraction (%).

### 2.4 Quantifying pairwise imbalance among ESs

Pairwise imbalance among ESs was evaluated using root mean square deviation (RMSD), which quantifies the degree to which paired services deviate from equal relative performance (Bradford and D’Amato, 2012; Zeng et al., 2019; Lan et al., 2023). RMSD describes differences in the standardized performance of paired services rather than the direction of their statistical association; therefore, it does not distinguish trade-offs from synergies.

Before RMSD calculation, all ES indicators were transformed to the 0–1 range using min–max normalization across all 36 plots. RMSD values were then calculated for all pairwise combinations of the eight ESs, yielding 28 service pairs, both across all plots and separately within each restoration pathway. Larger RMSD values indicate stronger imbalance between paired services, whereas smaller values indicate more similar relative performance. For descriptive comparisons, pairwise imbalance was classified as high (RMSD ≥ 0.50), intermediate (0.25 ≤ RMSD < 0.50), or low (RMSD < 0.25). These categories describe imbalance magnitude rather than statistical significance.

Service pairs are denoted using the abbreviations in **Table 1**, for example, WC–RC for water conservation and regeneration capacity. RMSD was calculated using Equation 7-1:

(7-1) $$RMSD=\sqrt{\frac{1}{n}\times \sum_{i=1}^{n}{\left(ES_{ij}-ES_{ik}\right)}^{2}}$$

where *ES_ij_* and *ES_ik_* are the min–max-normalized values of services *j* and *k* in plot *i*, respectively, and *n* is the number of plots included in the calculation (36 for the pooled analysis and 12 for each restoration pathway).

### 2.5 Quantifying ecosystem multifunctionality

EMF was quantified using both the averaging and threshold-based approaches (Byrnes et al., 2014; Costa et al., 2026; Lan et al., 2023). Before calculation, all ES indicators were transformed to the 0–1 range using the min–max normalization described in Section 2.4 to remove differences in units and numerical ranges among services. Under the averaging approach, EMF was calculated as the arithmetic mean of the eight standardized ESs, giving equal weight to each indicator and summarizing overall relative service performance at the plot scale (Equation 8-1):

(8-1) $$EMF-Mean=\frac{1}{F}\sum_{i=1}^{F}g(ESi)$$

where EMF-Mean denotes the multifunctionality index quantified using the averaging approach; *F* is the number of ESs measured (*F* = 8); *ES_i_* represents the measured value of ecosystem service *i*; and *g* denotes the normalization method.

Under the threshold-based approach, EMF was quantified as the number of ESs in each plot that reached or exceeded a given threshold. Three thresholds were used: 25%, 50%, and 75%, corresponding to EMF-T25, EMF-T50, and EMF-T75, respectively. These thresholds represent progressively higher levels of relative service performance on the normalized scale (Equation 8-2):

(8-2) $$EMF-Ti=\sum_{i=1}^{F}\text{I}(ES_{ij}^{std}\geq T)$$

where *T_i_* is 0.25, 0.50, or 0.75, and *I* is an indicator function equal to 1 when a service reaches or exceeds the threshold and 0 otherwise. Threshold-based EMF therefore ranges from 0 to 8. Combining the two approaches distinguished average service performance from the number of services maintained above increasingly demanding relative thresholds. Both approaches refer to the service dimensions represented by the indicators in **Table 1**.

### 2.6 Statistical analysis

Differences in each EMF metric among restoration pathways were assessed using one-way analysis of variance followed by Tukey’s *post hoc* test. Univariate linear models and forward stepwise regression were used for exploratory candidate-variable screening. Pre-screened predictors were evaluated separately for each restoration pathway and EMF metric using single-predictor linear mixed-effects models (LMMs) with a random intercept for region. Standardized coefficients, 95% confidence intervals, and *p*-values characterized region-adjusted associations.

Relative importance was quantified using the Lindeman–Merenda–Gold (LMG) decomposition of *R^2^* from standardized ordinary least-squares (OLS) multiple regression models. The same pathway-specific candidate set was used across all four EMF metrics. LMG contributions were evaluated using permutation tests. Screening criteria, candidate sets, and supplementary ES correlation methods are provided in Section S1.5.

An observed-variable covariance-based structural equation model (SEM) was fitted to all 36 plots using maximum likelihood estimation in lavaan (Rosseel, 2012). Each conceptual component was represented by a single observed indicator. Continuous variables were Z-standardized, and restoration pathway was numerically coded and treated as a continuous predictor in the SEM. Standardized direct, indirect, and total effects were evaluated using 5000 non-parametric bootstrap resamples with 95% confidence intervals.

The ecological framework, exploratory path modifications, and candidate-model comparisons are documented in Section S1.4 and **Supplementary Tables S5–S6**. Threshold-based SEMs retained the final EMF-Mean model’s upstream structure and substituted EMF-T25, EMF-T50, or EMF-T75 as the terminal response. Estimated pathways were interpreted as conditional associations.

All analyses used plots as the sampling units. Data processing used Excel 2021; statistical analysis and visualization used R 4.4.2, SPSS 26, and Origin 2021. Supplementary ES correlations were calculated in Python. Unless otherwise specified, values are reported as means ± standard deviations, with statistical significance assessed at *p* < 0.05.

## 3 Results

### 3.1 Patterns of pairwise ecosystem-service imbalance across restoration pathways

Pairwise RMSD values among the eight ESs ranged from 0.0825 to 0.7473 in the pathway-specific analysis (**Figure 2**). Intermediate imbalances were the most frequent category in pure plantations and secondary forests, whereas high imbalances were most frequent in mixed plantations.

**Figure 2:**
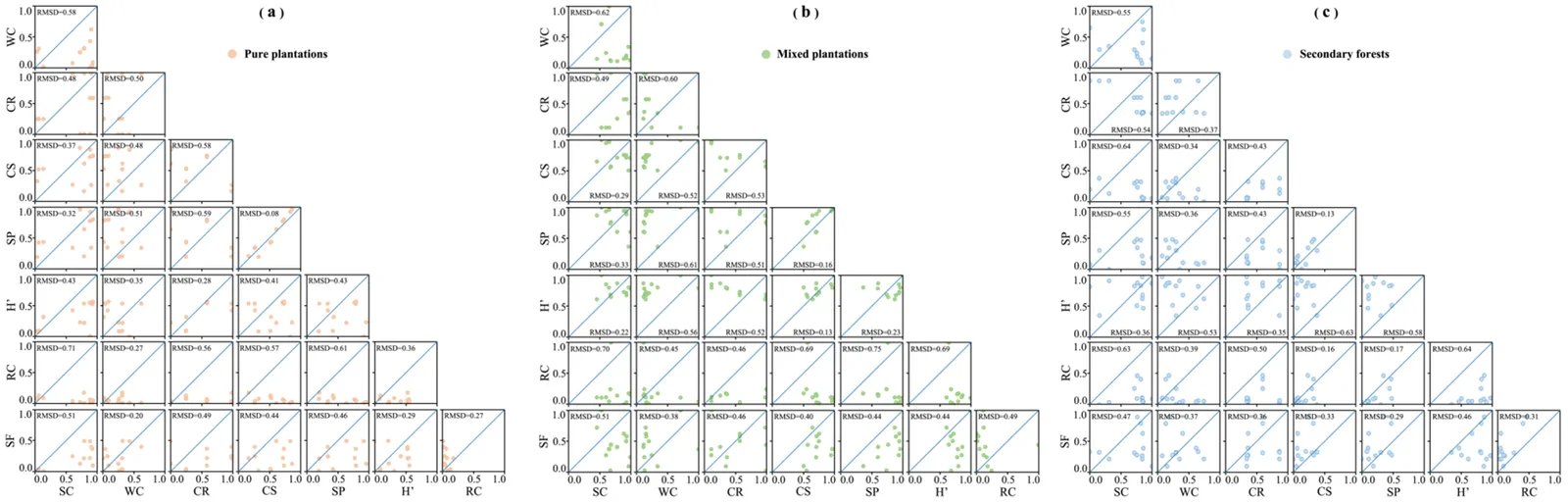
Pairwise ecosystem-service imbalances across restoration pathways. **(a)** Pure *C. lanceolata* plantations; **(b)** mixed plantations; **(c)** secondary forests. Points represent plots, and solid lines indicate equal normalized service values (1:1). Root mean square deviation (RMSD) quantifies pairwise imbalance: low (<0.25), intermediate (0.25–<0.50), or high (≥0.50), classified using unrounded values. RMSD is not a correlation coefficient. Service abbreviations are defined in **Table 1**.

In pure *C. lanceolata* plantations, 9, 17, and 2 service pairs were classified as having high, intermediate, and low imbalances, respectively (**Figure 2a**). The largest imbalances occurred for SC–RC, SP–RC, CR–SP, CR–CS, and SC–WC. CS–SP and WC–SF exhibited low imbalances. The occurrence of RC in several highly imbalanced pairs showed that its standardized values differed substantially from those of several regulating and provisioning services.

In mixed plantations, 13, 11, and 4 service pairs exhibited high, intermediate, and low imbalances, respectively (**Figure 2b**). Mixed plantations contained the largest number of highly imbalanced service pairs among the three restoration pathways. The largest imbalances occurred for SP–RC, SC–RC, CS–RC, H′–RC, and SC–WC, whereas CS–H′, CS–SP, SC–H′, and SP–H′ exhibited low imbalances. Thus, mixed plantations combined closely matched performance in some service pairs with pronounced differences in others.

In secondary forests, 10, 15, and 3 service pairs exhibited high, intermediate, and low imbalances, respectively (**Figure 2c**). The largest imbalances occurred for H′–RC, SC–CS, CS–H′, SC–RC, and SP–H′, whereas CS–SP, CS–RC, and SP–RC showed low imbalances. Intermediate imbalances predominated in secondary forests, although pronounced mismatches remained among ESs related to tree diversity, natural regeneration, carbon storage, and soil conservation.

Overall, none of the three restoration pathways exhibited uniformly low pairwise ES imbalance. Mixed plantations had the most highly imbalanced service pairs, whereas intermediate imbalances predominated in pure plantations and secondary forests. Supplementary ES correlations describe covariation separately from RMSD-based imbalance (**Supplementary Figure S6**; Section S2.4).

### 3.2 Ecosystem multifunctionality across restoration pathways

Mixed plantations had significantly higher EMF-Mean than pure plantations and secondary forests (*p* < 0.05; **Figure 3a**). Under the averaging approach, mixed plantations had the highest EMF-Mean value (0.559), followed by secondary forests (0.403) and pure *C. lanceolata* plantations (0.383). The latter two pathways did not differ significantly. Mixed plantations also had the highest mean numbers of ESs reaching the three thresholds, although differences were significant only for EMF-T50 and EMF-T75, not for EMF-T25.

**Figure 3:**
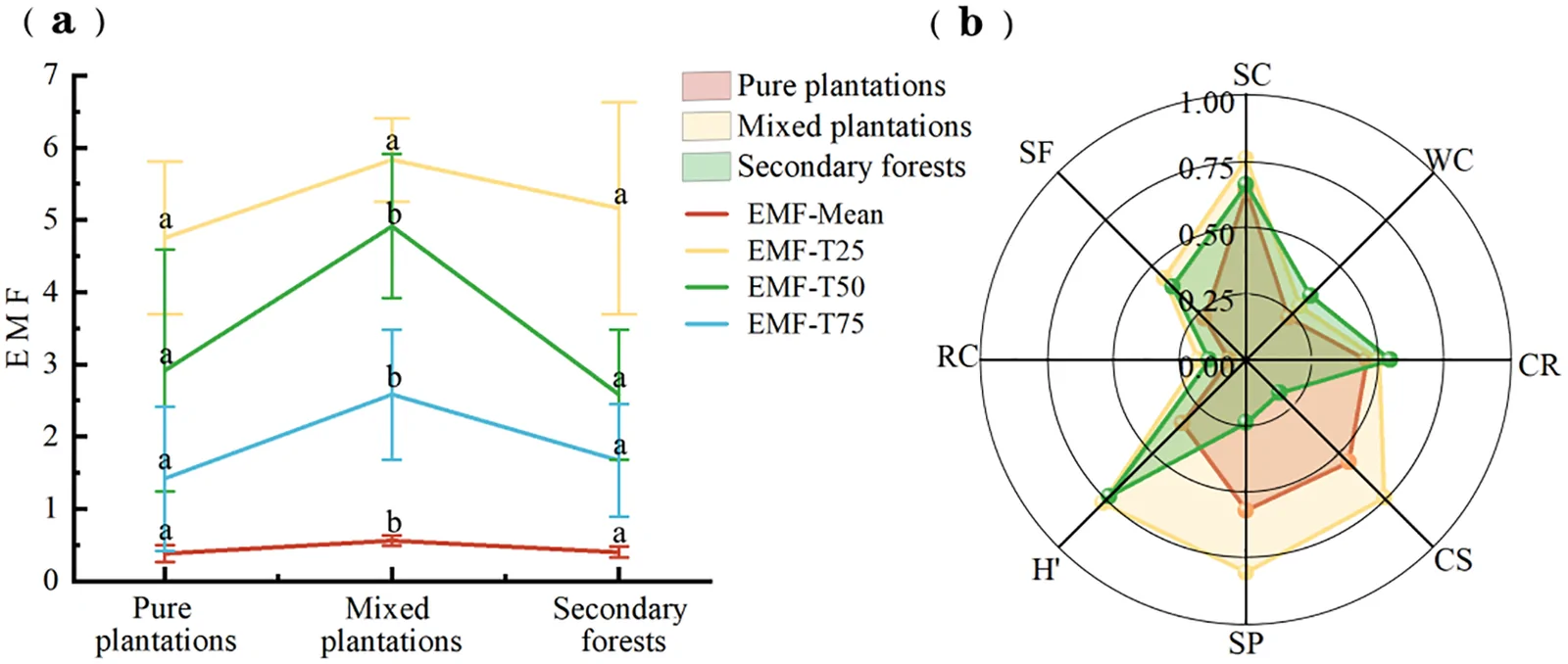
Ecosystem multifunctionality and service profiles across restoration pathways. **(a)** EMF-Mean and the numbers of services reaching the 25%, 50%, and 75% thresholds. Points and error bars show means ± standard deviations (*n* = 12 per pathway). Different lowercase letters indicate differences among pathways within each metric (one-way analysis of variance followed by Tukey’s test, *p* < 0.05). **(b)** Mean normalized service values. Service abbreviations are defined in **Table 1**.

The standardized ES profiles showed that restoration pathways differed in service composition as well as aggregate EMF (**Figure 3b**). Mixed plantations showed relatively high standardized values for SC, CR, CS, SP, and H′. Secondary forests showed relatively high values for SC, CR, and H′, whereas pure *C. lanceolata* plantations were characterized by relatively high SC, CS, and SP.

Higher aggregate EMF in mixed plantations was not accompanied by uniformly high values of all service indicators. RC remained low on the normalized scale across all three restoration pathways, whereas WC was higher in secondary forests than in either plantation type. These differences concerned the measured indicators of established sapling density and maximum litter–soil water-holding capacity, respectively. Mixed plantations therefore combined high aggregate multifunctionality with relatively low regeneration and water-retention indicators.

### 3.3 Candidate correlates of EMF

Exploratory univariate and forward stepwise regressions identified pathway-specific candidate variables (**Supplementary Figures S3–S4**; **Supplementary Table S8**). Their region-adjusted associations with EMF were evaluated using single-predictor LMMs (**Figure 4**).

**Figure 4:**
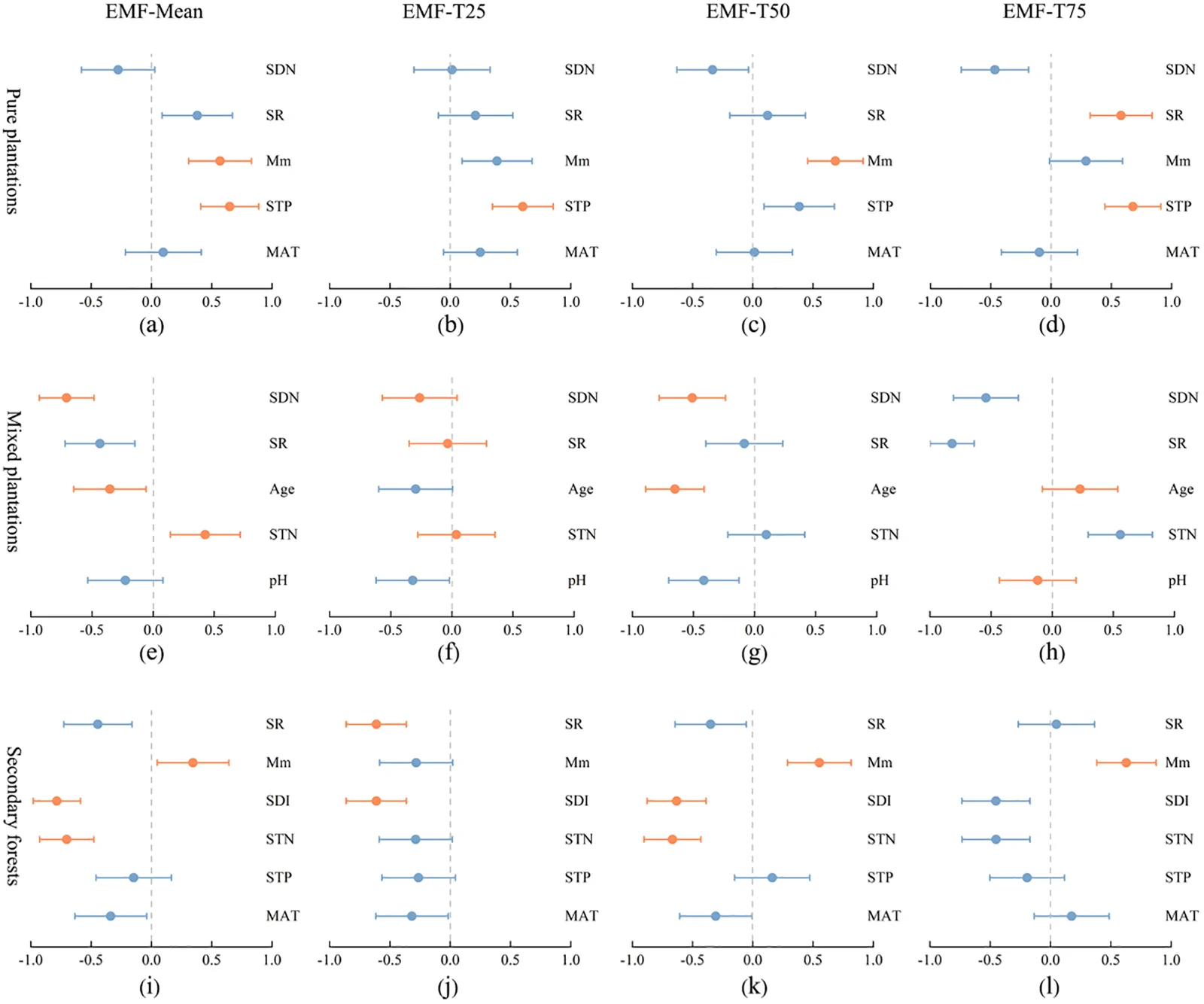
Standardized coefficients from single-predictor linear mixed-effects models. Points and bars show standardized coefficients and 95% confidence intervals from single-predictor LMMs with a random intercept for region. Orange indicates *p* < 0.05; blue indicates *p* ≥ 0.05. Dashed lines mark zero. Rows represent pure plantations, mixed plantations, and secondary forests; columns represent EMF-Mean, EMF-T25, EMF-T50, and EMF-T75. Abbreviations follow Section 2.2.

Associations between candidate variables and EMF varied among restoration pathways and thresholds. In pure *C. lanceolata* plantations, STP and M_m_ were generally positively associated with EMF. SR was positively associated with high-threshold EMF, whereas SDN showed negative associations with EMF-T50 and EMF-T75. In mixed plantations, SDN and SR generally had negative coefficients, while the association with Age varied among EMF metrics and was negative for EMF-Mean and EMF-T50. In secondary forests, SDI and STN were mostly negatively associated with EMF, whereas M_m_ showed positive associations, especially at intermediate and high thresholds. The direction and statistical support of individual associations varied among restoration pathways and EMF metrics.

LMG decomposition of the full candidate OLS models quantified each predictor’s share of explained variance (**Figure 5**). For EMF-Mean, M_m_ and STP accounted for the largest shares of explained variance in pure plantations (37.10% and 33.94%, respectively). In mixed plantations, SDN had the highest relative importance (34.04%), followed by Age (22.83%). In secondary forests, SDI and STN contributed the largest shares (38.37% and 27.54%, respectively). Rankings varied across threshold-based metrics: Age ranked highest for EMF-T50 and SR for EMF-T75 in mixed plantations, whereas SR, STN, and M_m_ ranked highest for EMF-T25, EMF-T50, and EMF-T75, respectively, in secondary forests. Because Age varied within a restricted range, its relative-importance value describes variation only among the sampled middle-aged stands and does not provide evidence of a general age-dependent change in EMF.

**Figure 5:**
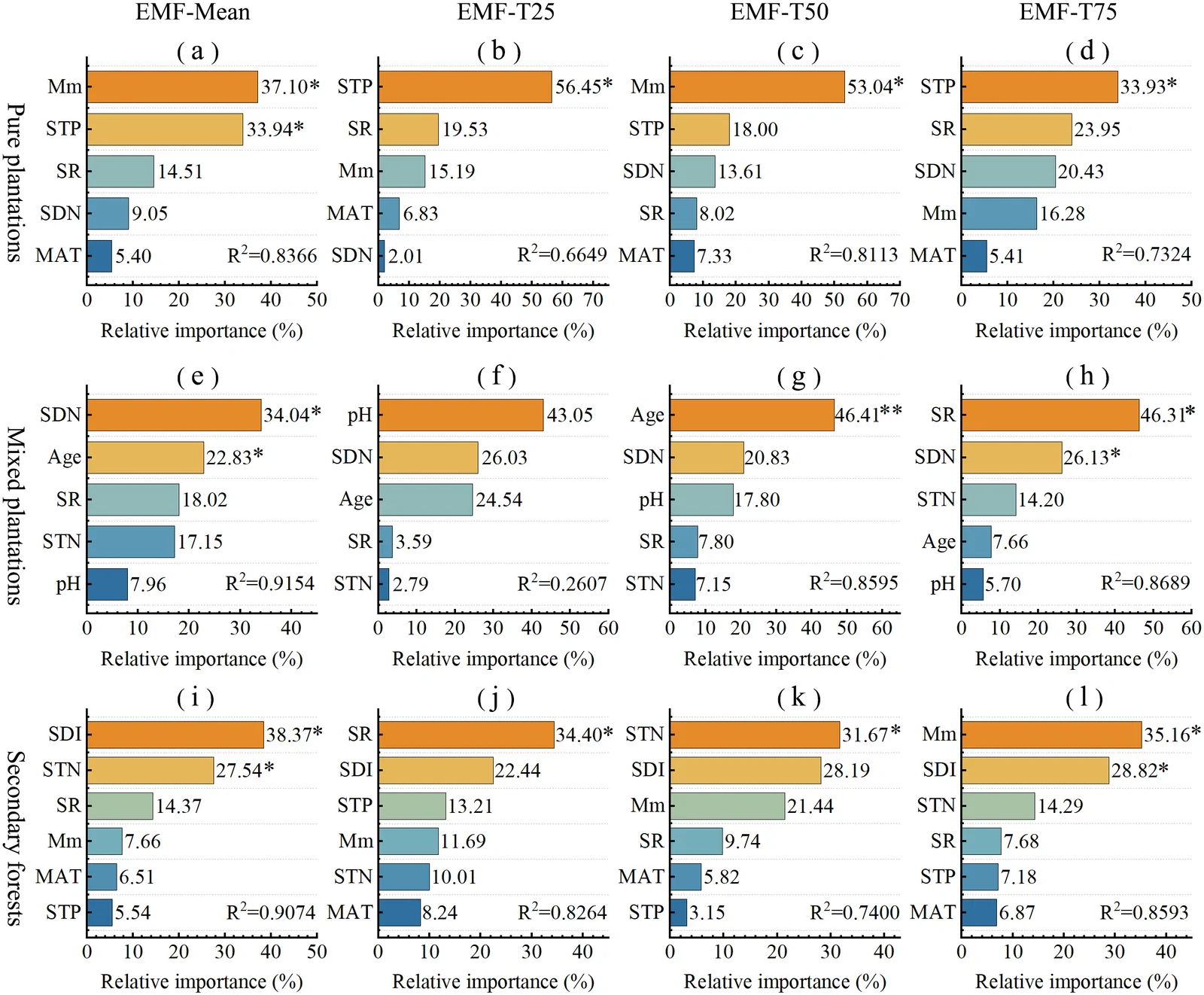
Relative importance of candidate variables for ecosystem multifunctionality. Bars show percentage contributions to model *R^2^* from LMG decomposition of multiple OLS regressions. Asterisks indicate permutation-test significance of LMG contributions (199 permutations; *p < 0.05, **p< 0.01). Panel arrangement and abbreviations follow **Figure 4**.

Overall, the regression associations and relative-importance rankings differed among restoration pathways and EMF metrics. Soil phosphorus and species mingling were prominent correlates in pure *C. lanceolata* plantations, structural and compositional variables were prominent in mixed plantations, and stand density and soil nitrogen accounted for the largest shares of explained EMF-Mean variation in secondary forests. Although MAT contributed to explained variation in some models, its associations were not consistent across EMF metrics.

### 3.4 SEM-based pathways linking soil, stand structure, diversity, and EMF

The final SEM incorporated restoration pathway, soil nutrient status, species diversity, stand structure, spatial stand structure, and structural diversity. Soil nutrient status, species diversity, stand structure, spatial stand structure, structural diversity, and multifunctionality were represented by STP, SR, SDI, Mm, SDN, and EMF-Mean, respectively. The selected model had χ^2^ = 5.263 with 6 degrees of freedom (*p* = 0.511; χ^2^/df = 0.877; goodness-of-fit index [GFI] = 0.956) and explained 68.2% of the variation in EMF-Mean (**Figure 6**). The candidate-model comparisons are presented in **Supplementary Table S6**.

**Figure 6:**
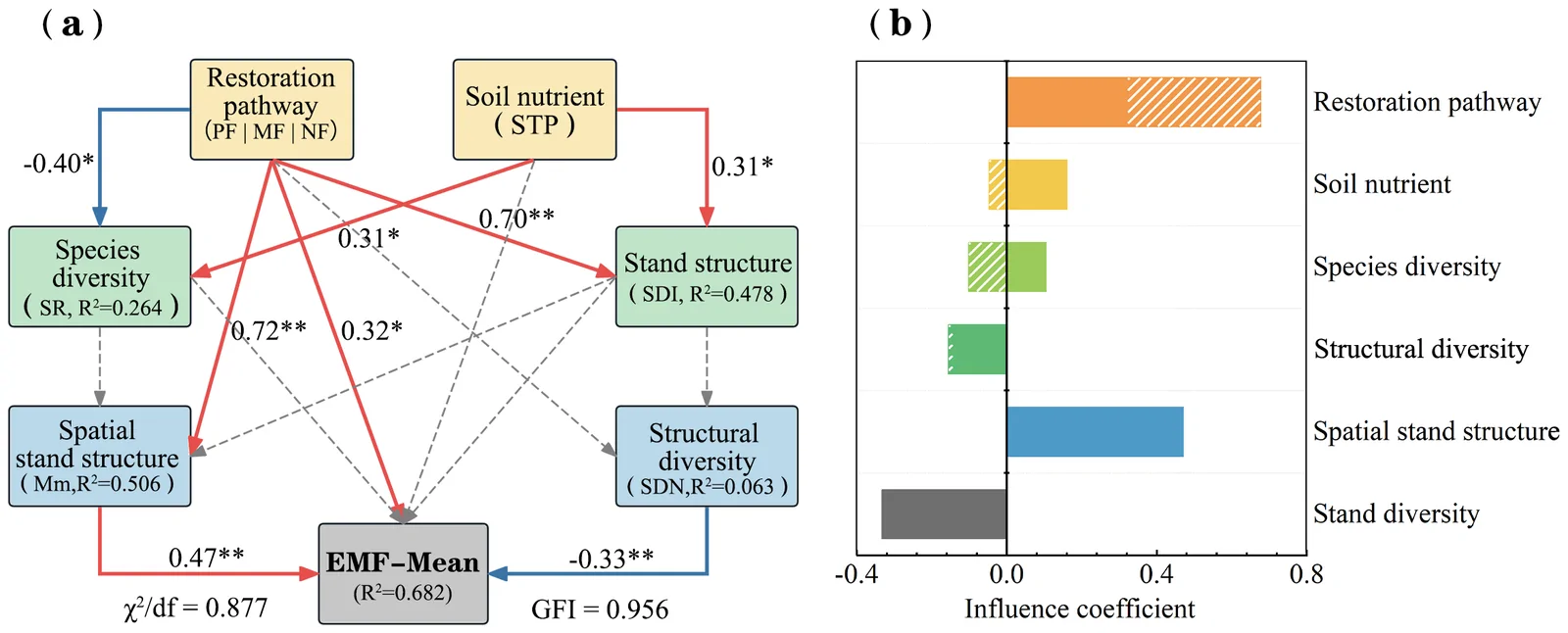
Direct and indirect associations with ecosystem multifunctionality in the final SEM. **(a)** Standardized path coefficients and explained variance (*R^2^*). Red and blue solid arrows indicate significant positive and negative associations, respectively (*p < 0.05; **p < 0.01); gray dashed arrows indicate nonsignificant paths. **(b)** Standardized direct effects (solid bars) and summed indirect effects (hatched bars). GFI, goodness-of-fit index; df, degrees of freedom. Predictor abbreviations are defined in Section 2.2. Arrows do not establish causation.

Restoration pathway showed positive direct and summed indirect associations with EMF-Mean, with a standardized direct coefficient of 0.322. Soil nutrient status was positively associated with stand structure (β = 0.314, *p* = 0.024) and species diversity (*p* < 0.05).

Among the direct pathways to EMF-Mean, spatial stand structure showed the strongest positive standardized association (β = 0.472, *p* = 0.005), whereas structural diversity showed a significant negative standardized association (β = −0.333, *p* = 0.009). In contrast, the direct coefficients for soil nutrient status, species diversity, and stand structure were smaller in magnitude and were not statistically significant. The positive summed indirect estimate for restoration pathway indicated that its association with EMF-Mean also involved the modeled community and structural attributes (**Figure 6**).

Threshold-based SEMs explained 20.5%, 67.2%, and 54.7% of the variation in EMF-T25, EMF-T50, and EMF-T75, respectively. No direct path to EMF-T25 was significant, whereas positive M_m_ and negative SDN associations persisted at the 50% and 75% thresholds (**Supplementary Figure S5**; Section S2.3).

## 4 Discussion

### 4.1 Pathway-specific ecosystem service bundles and relative functional bottlenecks

Intermediate pairwise imbalance was the most frequent category in pure plantations and secondary forests, whereas high imbalance was the most frequent category in mixed plantations. RMSD quantifies disparity between standardized service values rather than covariation and therefore does not establish statistical trade-offs or synergies. Low RMSD may reflect similarly high or low service levels. The observed differences in service profiles are consistent with evidence that forest composition, management history, and restoration strategy shape the joint supply of multiple services (Cortés-Calderón et al., 2021; Xiong et al., 2023). Individual services also depend on different resource pools and ecological processes, so improvement in one function need not be matched by comparable gains in others (Bennett et al., 2009; Raudsepp-Hearne et al., 2010).

Regeneration capacity remained relatively low across all three pathways and frequently formed highly imbalanced service pairs. Tree recruitment depends on propagule availability, understory light, microsite conditions, and competition, which may not align with the conditions favoring biomass accumulation (Luo et al., 2025; Pan et al., 2022). In a pot experiment, thin to moderate litter cover favored *C. lanceolata* seedling establishment, whereas excessive cover inhibited establishment despite improved moisture retention (Liu et al., 2026). These contrasting responses illustrate how forest-floor conditions can affect regulating functions and regeneration differently, providing a plausible explanation for uneven service provision.

Water conservation (WC) was relatively low in pure and mixed plantations and higher in secondary forests. Because mixed plantations also contained broadleaved species, the presence of broadleaved trees alone cannot explain this contrast. Litter accumulation and decomposition can alter forest-floor water storage, while root distribution and turnover can influence organic matter inputs and soil pore development. A comparable subtropical study reported higher soil water-holding capacity in secondary forests than in *C. lanceolata* plantations, with positive associations with soil porosity and negative associations with bulk density (Zhou et al., 2025). These processes offer plausible explanations for the WC contrast, although their contributions were not separated here. This comparison concerns maximum storage capacity; water-yield responses additionally involve hydrological fluxes and catchment conditions (Filoso et al., 2017; Schwärzel et al., 2020; Zhu et al., 2024).

Pure *C. lanceolata* plantations showed a relatively specialized service profile, with comparatively strong soil conservation, carbon storage, and stand productivity but pronounced imbalances involving regeneration capacity. This profile is consistent with their production-oriented management legacy. Soil-mediated mechanisms are also plausible given the physicochemical, microbial, and enzymatic differences reported for *C. lanceolata* monocultures (Cortés-Calderón et al., 2021; Guo et al., 2022; Xiong et al., 2023).

Mixed plantations combined the highest aggregate EMF with the largest number of highly imbalanced service pairs. Such uneven provision is consistent with differing associations of forest composition and structure with individual services (Felipe-Lucia et al., 2018; Gamfeldt et al., 2013; Liu et al., 2018). Secondary forests exhibited a different service combination, including relatively higher WC, although their EMF-Mean did not differ significantly from that of pure plantations. These contrasting profiles are consistent with evidence that service provision varies with tree functional groups and restoration stage (Zeng et al., 2019), and that multifunctionality recovery depends on restoration strategy, forest type, and recovery time (Cruz-Alonso et al., 2019).

Consistent with H1 and H3, service bundles differed among pathways, regeneration capacity remained relatively low, and static water-retention shortfalls were less pronounced in secondary forests. Aggregate EMF and service balance provide complementary information: high multifunctionality can coexist with pronounced disparities among individual services.

### 4.2 Mixed plantations combine higher multifunctionality with persistent service imbalance

Mixed plantations had significantly higher EMF-Mean, EMF-T50, and EMF-T75 than the other pathways, whereas their numerical advantage in EMF-T25 was not statistically significant. Their advantage was evident in both mean service performance and the number of services reaching the 50% and 75% thresholds. The distinction matters because averaging and threshold-based approaches emphasize different aspects of multifunctionality: the former summarizes mean standardized performance, whereas the latter records how many services exceed a specified threshold (Manning et al., 2018; Zeng et al., 2021). These thresholds describe performance relative to the sampled stands rather than externally defined ecological requirements.

Maintaining multiple functions generally requires a broader range of biotic attributes than maintaining any single function because different species and stand components contribute to different ecological processes (Gross et al., 2017; Hector and Bagchi, 2007). The sampled mixtures included contrasting broadleaved functional types: in Enshi, *C. lanceolata* occurred with deciduous *Quercus serrata* and evergreen *Q. glauca*; in Longnan, mixtures included evergreen *Castanopsis carlesii* and deciduous *Nyssa sinensis* (**Supplementary Table S1**). Differences in foliage phenology and crown architecture provide opportunities for complementary light use across seasons and canopy positions. This interpretation is consistent with research linking functional diversity to multifunctionality in subtropical plantations and with evidence that plant traits are associated with different service responses (Li et al., 2022; Pan et al., 2022).

Thinning followed by broadleaved enrichment may have allowed retained *C. lanceolata* trees and later-established cohorts to occupy different canopy positions, improving crown packing and resource capture (Liu et al., 2018). Canopy development contributes to the CR proxy and the vegetation-cover component of modeled SC, while accumulated biomass and timber volume underpin CS and SP. Greater canopy occupation may nevertheless restrict light availability for regeneration, and improvements in canopy-related services need not be accompanied by proportional increases in litter–soil water storage. These are plausible mechanisms; vertical stratification and resource partitioning were not directly tested.

Higher aggregate EMF and pronounced pairwise imbalance are compatible because they describe different properties of service provision. Relatively strong SC, CR, CS, SP, and tree species diversity can raise aggregate EMF while lower RC and WC generate pronounced disparities. The multifunctionality of species mixtures depends on species identity, relative abundance, stand structure, and the particular functions evaluated (Felipe-Lucia et al., 2018; Gamfeldt et al., 2013; Li et al., 2024b). Functional complementarity may therefore broaden service provision without equalizing all components of the service bundle.

The distinct profiles of pure plantations and secondary forests further illustrate why restoration performance depends on the service dimensions considered (Himes et al., 2020). These results support H2: mixed plantations achieved higher aggregate EMF without consistently lower service imbalance. The comparison applies to the sampled 32–39-year-old stands and does not establish that species mixing alone caused the observed differences.

### 4.3 Pathway-specific correlates of multifunctionality across thresholds

No single measured attribute was consistently associated with EMF across all restoration pathways, metrics, and thresholds. The differing associations identified by the regression models and SEM are consistent with the context dependence of forest multifunctionality (Costa et al., 2026; Sanaei et al., 2021). They also indicate the importance of distinguishing species composition, neighborhood arrangement, stand crowding, and diameter-class diversity rather than treating them as interchangeable measures of structural complexity.

In the SEM, mingling degree had a positive association with EMF-Mean, whereas diameter-based structural diversity had a negative association. Mingling describes the interspersion of different species within local neighborhoods, where species identity can influence competition and complementarity (Fichtner et al., 2017). Simulations parameterized with measurements from the Biodiversity–Ecosystem Functioning Experiment China (BEF-China) indicate that species arrangement can influence biomass production and the spatial distribution and decomposition of litter (Beugnon et al., 2025). SDN, by contrast, describes the distribution of individuals among diameter classes; it does not directly measure vertical canopy stratification or competitive asymmetry. Studies have reported benefits of structural diversity for subtropical forest multifunctionality (Ouyang et al., 2023), but negative associations with productivity have also been observed in young tree diversity experiments (Jensen et al., 2025). Differences in structural metrics, stand development, and the functions evaluated can therefore help explain contrasting associations (Forrester, 2019).

In pure *C. lanceolata* plantations, soil total phosphorus (STP) and mingling degree (M_m_) were generally positively associated with EMF. The phosphorus association is consistent with the importance of nutrient conditions in highly weathered subtropical soils and with evidence linking stand conditions to phosphorus fractions and tree nutrient status (Fang et al., 2017; Zhu et al., 2021). However, STP represents the broader soil phosphorus pool rather than plant-available phosphorus, so its positive association with EMF does not demonstrate that phosphorus addition would enhance multifunctionality. The association with M_m_ also implicates local species arrangement, although complementary resource use was not measured directly.

Within mixed plantations, the predominantly negative associations involving SDN and SR do not contradict their higher EMF relative to the other pathways. A difference between restoration pathways need not be reproduced by a richness gradient within one pathway, where species identity, abundance, and stand development also vary. Functional composition can therefore be more informative than species number alone when interpreting mixture performance (Li et al., 2022, 2024b). Likewise, the negative associations of stand age with EMF-Mean and EMF-T50 were restricted to the 32–39-year age range and were not consistent across all thresholds. They cannot establish a general decline in multifunctionality as mixed plantations mature.

In secondary forests, the negative associations of SDI with EMF are compatible with greater resource competition in more crowded stands, although competition was not demonstrated as the mediating process (Ma et al., 2025). The positive associations with M_m_ at intermediate and high thresholds suggest that neighborhood composition may be relevant alongside stand crowding. The negative STN association should be interpreted as a conditional relationship within these stands: total soil nitrogen can covary with organic matter accumulation, species composition, and decomposition, and does not directly indicate nitrogen availability or nutrient imbalance. Such dependence on stand development and above- and belowground conditions is consistent with research on secondary-forest multifunctionality (Matsuo et al., 2026).

Threshold dependence further qualifies these interpretations. No direct path to EMF-T25 was significant in the threshold-based SEM, whereas the positive M_m_ and negative SDN associations persisted at the 50% and 75% thresholds. These results are consistent with H4: associations with M_m_ and SDN were statistically supported at the 50% and 75% thresholds, whereas no direct association was significant at the 25% threshold. Threshold choice changes which aspects of joint service provision are emphasized (Byrnes et al., 2014; van der Plas et al., 2016). Because the threshold models used the same plots, service indicators, and upstream path structure, they assessed whether associations persisted under alternative EMF definitions; they did not independently validate the proposed mechanisms.

### 4.4 Management implications and study limitations

Management should address pathway-specific bottlenecks rather than attempt to maximize a single ecosystem service (Pukkala, 2016; Schwaiger et al., 2019). Because the present evidence is observational, the following measures should be regarded as candidate strategies. In pure *C. lanceolata* plantations, moderate density regulation, retention of naturally regenerated broadleaved trees, and protection of forest-floor litter and understory vegetation may reduce structural homogeneity and support regeneration (Huang et al., 2024; Li et al., 2021, 2022; Yang et al., 2023; Yu et al., 2022). In mixed plantations, management could emphasize functionally complementary species, appropriate species proportions, and neighborhood arrangement rather than increases in richness alone (Beugnon et al., 2025; Stritih et al., 2026). In secondary forests, retaining native trees, seed sources, and established regeneration could support continued recovery, with localized tending considered where excessive crowding is confirmed (Deng et al., 2023; Elliott et al., 2023; Robinson et al., 2025; Yang et al., 2023). The contrasting service profiles may also inform restoration planning where management objectives differ (Hua et al., 2022; Pukkala, 2016).

Inference is restricted to 36 observational plots across four subtropical regions, with three plots per restoration pathway in each region and stand ages of 32–39 years. This restricted range improved developmental comparability among pathways but did not eliminate residual stand-development effects. Age was associated with several EMF metrics in mixed plantations, but the narrow range and limited replication precluded a robust test of the restoration pathway × age interaction. Some age dependence of the pathway contrasts therefore cannot be excluded. This cross-sectional comparison of middle-aged stands cannot establish whether pathway differences persist, converge, or reverse at other developmental stages. Similarly, the LMMs, relative-importance analysis, and SEM identify statistical associations and plausible pathways rather than causal mechanisms. Future chronosequences, repeated censuses, and targeted experiments should test the temporal stability of these relationships and incorporate functional traits, regeneration processes, root dynamics, soil microbial communities, and seasonal hydrological dynamics.

Service comparisons were limited to the biophysical dimensions represented by the selected indicators (**Table 1**). Specifically, CR represented canopy-based microclimate-buffering potential because understory temperature and humidity were not measured directly; WC quantified static litter–soil water-storage capacity rather than runoff regulation or groundwater recharge; and SF represented only the SOC-related organic-matter component of fertility, excluding nutrient availability and cation exchange capacity (Lal, 2016). Some indicators also contained overlapping information: CS and SP were strongly positively correlated in the pooled data (**Supplementary Figure S6**), consistent with their shared dependence on standing stocks. Aggregate EMF should therefore be interpreted alongside individual service profiles.

## 5 Conclusions

Across the sampled 32–39-year-old stands, the three restoration pathways were associated with distinct ecosystem-service bundles. Mixed plantations had significantly higher EMF-Mean, EMF-T50, and EMF-T75 than pure *C. lanceolata* plantations and secondary forests, but also contained the largest number of highly imbalanced service pairs. Higher aggregate EMF therefore did not necessarily correspond to a more balanced service bundle. Regeneration capacity remained relatively low across all pathways, whereas static water retention was lower in pure and mixed plantations than in secondary forests.

Correlates of EMF varied among pathways and thresholds, with spatial mingling and diameter-based structural diversity showing contrasting associations. Restoration assessments should therefore consider both aggregate EMF and the relative performance of individual services. These findings can inform pathway-specific management priorities that address relatively underperforming services while retaining existing functional strengths. As an observational comparison of middle-aged forests, this study does not establish causal responses to restoration treatments or determine whether the observed differences persist across stand-development stages.

## Data Availability

The raw data supporting the conclusions of this article will be made available by the authors, without undue reservation.
