# Supplementary material for: Restoration pathways reshape forest structure and multifunctionality in subtropical plantations and secondary forests
Source: Front Plant Sci. 2026 Oct 21;17:1941032. doi: 10.3389/fpls.2026.1941032 (PMC13731253; doi:10.3389/fpls.2026.1941032)
Supplement: Supplementary file 1 [file DataSheet1.docx]

## Supporting Information

### S1 Methodological Supplement

#### S1.1 Basic characteristics of the sample plots

The study included 36 plots representing three restoration pathways: pure *Cunninghamia lanceolata* plantations (PF), *C. lanceolata*–broadleaved mixed plantations (MF), and naturally regenerated secondary forests (NF). All sampled stands were 32–39 years old. Prior to the systematic plot survey, slope, aspect, geographic coordinates, and elevation were recorded at the center of each plot (Figure S1a). Detailed site information is provided in Table S1.

Table S1. Site characteristics of the sampled plots

| **Plot No.** | **Site** | **Stand type** | **Dominant species** | **Longitude (E)** | **Latitude (N)** | **Elevation (m)** | **Slope gradient (°)** | **Slope direction** | **Soil type** |
| --- | --- | --- | --- | --- | --- | --- | --- | --- | --- |
| 1 | ES | NF | *C. lanceolata*, *B. polycarpa* | 109.434 | 30.371 | 1248.6 | 19 | W | Y |
| 2 | ES | NF | *C. lanceolata*, *B. polycarpa* | 109.433 | 30.373 | 1213.4 | 16 | W | Y |
| 3 | ES | NF | *C. lanceolata*, *B. polycarpa* | 109.433 | 30.373 | 1206.01 | 19 | W | Y |
| 4 | ES | PF | *C. lanceolata* | 109.423 | 30.561 | 1173.92 | 27 | NW | Y |
| 5 | ES | PF | *C. lanceolata* | 109.423 | 30.562 | 1208.18 | 42 | NW | Y |
| 6 | ES | PF | *C. lanceolata* | 109.423 | 30.562 | 1200.67 | 36 | NW | Y |
| 7 | ES | MF | *C. lanceolata*, *Q. serrata, Q. glauca* | 109.424 | 30.331 | 1306.01 | 46 | SW | Y |
| 8 | ES | MF | *C. lanceolata*, *Q. serrata*, *Q. glauca* | 109.424 | 30.331 | 1306.01 | 34 | S | Y |
| 9 | ES | MF | *C. lanceolata*, *Q. serrata*, *Q. glauca* | 109.424 | 30.331 | 1306.01 | 32 | S | Y |
| 10 | HT | NF | *C. lanceolata*, *Q. glauca*- *M. pauhoi* | 109.610 | 26.853 | 372.89 | 38 | S | Y-B |
| 11 | HT | NF | *C. lanceolata*, *Q. glauca*, *M. pauhoi* | 109.609 | 26.853 | 385.99 | 36 | S | Y-B |
| 12 | HT | NF | *C. lanceolata*, *Q. glauca*, *M. pauhoi* | 109.610 | 26.854 | 358.99 | 34 | S | Y-B |
| 13 | HT | PF | *C. lanceolata*, *P. laurocerasus* | 109.607 | 26.849 | 470.3 | 16 | SW | R |
| 14 | HT | PF | *C. lanceolata*, *P. laurocerasus* | 109.607 | 26.849 | 454.8 | 16 | SW | R |
| 15 | HT | PF | *C. lanceolata*, *P. laurocerasus* | 109.607 | 26.849 | 451 | 14 | S | R |
| 16 | HT | MF | *C. Lanceolata*, *Q. glauca*, *D. oldhamii* | 109.602 | 26.851 | 416.69 | 14 | E | R |
| 17 | HT | MF | *C. lanceolata*, *Q. glauca*, *D. oldhamii* | 109.602 | 26.851 | 419.79 | 25 | SE | R |
| 18 | HT | MF | *C. lanceolata*, *Q. glauca*, *D. oldhamii* | 109.602 | 26.851 | 422.5 | 18 | S | R |
| 19 | PX | NF | *C. lanceolata*, *F. langkokensis* | 106.704 | 22.304 | 433.57 | 29 | S | Y |
| 20 | PX | NF | *C. lanceolata*, *F. langkokensis* | 106.704 | 22.304 | 418.07 | 28 | NW | Y |
| 21 | PX | NF | *C. lanceolata*, *F. langkokensis* | 106.704 | 22.304 | 429.84 | 29 | NW | Y |
| 22 | PX | PF | *C. lanceolata* | 106.859 | 22.032 | 443.16 | 35 | E | Y |
| 23 | PX | PF | *C. lanceolata* | 106.860 | 22.032 | 434.38 | 19 | SE | Y |
| 24 | PX | PF | *C. lanceolata* | 106.860 | 22.032 | 460.62 | 16 | SE | Y |
| 25 | PX | MF | *C. lanceolata*, *Q. griffithii*, *E. fordii* | 106.855 | 22.053 | 457.44 | 48 | SW | Y |
| 26 | PX | MF | *C. lanceolata*, *Q. griffithii*, *E. fordii* | 106.855 | 22.054 | 409.3 | 27 | SW | Y |
| 27 | PX | MF | *C. Lanceolata*, *Q. griffithii*, *E. fordii* | 106.855 | 22.055 | 423.4 | 28 | SW | Y |
| 28 | LN | NF | *C. lanceolata*, *S. superba*, *L. formosana* | 114.443 | 24.582 | 413.2 | 4 | S | Y-R |
| 29 | LN | NF | *C. lanceolata*, *S. superba*, *L. formosana* | 114.443 | 24.582 | 412.3 | 10 | S | Y-R |
| 30 | LN | NF | *C. lanceolata*, *S. superba*, *L. formosana* | 114.443 | 24.582 | 407.1 | 10 | S | Y-R |
| 31 | LN | PF | *C. lanceolata* | 114.478 | 24.548 | 400.46 | 30 | SE | Y-R |
| 32 | LN | PF | *C. lanceolata* | 114.479 | 24.548 | 388.4 | 30 | S | Y-R |
| 33 | LN | PF | *C. lanceolata* | 114.479 | 24.548 | 396.14 | 30 | S | Y-R |
| 34 | LN | MF | *Cunninghamia lanceolata*, *Castanopsis carlesii*, *N. sinensis* | 114.451 | 24.543 | 406.7 | 10 | S | Y-R |
| 35 | LN | MF | *Cunninghamia lanceolata*, *Castanopsis carlesii*, *N. sinensis* | 114.455 | 24.589 | 405 | 5 | S | Y-R |
| 36 | LN | MF | *Cunninghamia lanceolata*, *Castanopsis carlesii*, *N. sinensis* | 114.451 | 24.592 | 378.8 | 3 | S | Y-R |

Note: In the table, ES, HT, PX, and LN denote Enshi, Huitong, Pingxiang, and Longnan, respectively. For stand type, PF denotes pure C. lanceolata plantations, MF denotes *C. lanceolata*–broadleaved mixed plantations, and NF denotes naturally regenerated secondary forests. For aspect, E, SE, S, SW, W, and NW indicate east, southeast, south, southwest, west, and northwest, respectively. For soil type, Y, Y-B, R, and Y-R represent yellow soil, yellow-brown soil, red soil, and yellow-red soil, respectively.

The three restoration pathways differed in their establishment and early silvicultural histories. PF consisted of artificially established stands dominated by *C. lanceolata* and received a single thinning operation at a stand age of 8–10 years. MF originated from planted *C. lanceolata* stands that were thinned at 8–10 years of age and subsequently enriched with broadleaved tree species. NF developed through natural succession following the clear-cutting of former *C. lanceolata* plantations, without subsequent tree planting. After these pathway-defining interventions, no further tending, thinning, enrichment planting, or other silvicultural treatments were applied in any of the selected stands. Stand characteristics, regeneration sources, and available management histories are summarized by region and restoration pathway in Table S2.

Table S2. Stand characteristics, regeneration sources, and silvicultural histories of the three restoration pathways across four study regions

| **Site** | **Stand type** | **No. of plots** | **Stand age (yr)** | **Current stand density (stems·ha^-1^)** | **Regeneration source** | **Major intervention** | **Management history** |
| --- | --- | --- | --- | --- | --- | --- | --- |
| ES | NF | 3 | 36 | 2016.75 ± 253.00 | Natural regeneration following the clear-cutting of a former *C. lanceolata* plantation | Clear-cutting followed by natural succession, without subsequent tree planting | No further tending or other silvicultural treatment |
| ES | PF | 3 | 36 | 2208.25 ± 212.50 | Artificial planting of *C. lanceolata* | A single tending thinning approximately 8 years after stand establishment | No further tending or other silvicultural treatment |
| ES | MF | 3 | 38 | 3241.75 ± 415.50 | Artificial planting of *C. lanceolata*, followed by enrichment planting of broadleaved species | A single tending thinning approximately 8 years after stand establishment, followed by enrichment planting of broadleaved tree species | No further tending or other silvicultural treatment |
| HT | NF | 3 | 32 | 2275.00 ± 209.00 | Natural regeneration following the clear-cutting of a former *C. lanceolata* plantation | Clear-cutting followed by natural succession, without subsequent tree planting | No further tending or other silvicultural treatment |
| HT | PF | 3 | 36 | 2258.25 ± 205.75 | Artificial planting of *C. lanceolata* | A single tending thinning approximately 10 years after stand establishment | No further tending or other silvicultural treatment |
| HT | MF | 3 | 34 | 2916.75 ± 351.25 | Artificial planting of *C. lanceolata*, followed by enrichment planting of broadleaved species | A single tending thinning approximately 10 years after stand establishment, followed by enrichment planting of broadleaved tree species | No further tending or other silvicultural treatment |
| PX | NF | 3 | 37 | 2050.00 ± 132.25 | Natural regeneration following the clear-cutting of a former *C. lanceolata* plantation | Clear-cutting followed by natural succession, without subsequent tree planting | No further tending or other silvicultural treatment |
| PX | PF | 3 | 33 | 2216.75 ± 289.75 | Artificial planting of *C. lanceolata* | A single tending thinning approximately 10 years after stand establishment | No further tending or other silvicultural treatment |
| PX | MF | 3 | 33 | 2500.00 ± 164.00 | Artificial planting of *C. lanceolata*, followed by enrichment planting of broadleaved species | A single tending thinning approximately 10 years after stand establishment, followed by enrichment planting of broadleaved tree species | No further tending or other silvicultural treatment |
| LN | NF | 3 | 32 | 2200.00 ± 248.00 | Natural regeneration following the clear-cutting of a former *C. lanceolata* plantation | Clear-cutting followed by natural succession, without subsequent tree planting | No further tending or other silvicultural treatment |
| LN | PF | 3 | 38 | 2366.75 ± 197.50 | Artificial planting of *C. lanceolata* | A single tending thinning approximately 8 years after stand establishment | No further tending or other silvicultural treatment |
| LN | MF | 3 | 39 | 2608.25 ± 177.25 | Artificial planting of *C. lanceolata*, followed by enrichment planting of broadleaved species | A single tending thinning approximately 8 years after stand establishment, followed by enrichment planting of broadleaved tree species | No further tending or other silvicultural treatment |

Note: Current stand density is presented as the mean ± standard deviation of three plots per region and restoration pathway. Stand age was determined from historical records of plantation establishment or recovery initiation and cross-validated against the mean age of sampled canopy trees. For PF and MF, stand age represents the time since the establishment of the original *C. lanceolata* plantation; for NF, it represents the time since the initiation of natural recovery following clear-cutting. Current stand density was calculated using the same tree-inclusion criterion as that applied in the plot census. For stand type, PF denotes pure *C. lanceolata* plantations, MF denotes *C. lanceolata*–broadleaved mixed plantations, and NF denotes naturally regenerated secondary forests.

#### S1.2 Survey methods

##### S1.2.1 Tree and shrub survey

In 2025, we conducted a comprehensive census of all study plots. All woody individuals with a diameter at breast height (*DBH*) ≥ 4 cm were tagged with uniquely numbered aluminum labels and marked with red paint at 1.3 m above the ground. After species identification to the lowest feasible taxonomic level, we recorded the *DBH*, total height (*H*), crown width (*C*), and the relative coordinates of each stem within the plot. For woody plants branching below breast height, stems reaching *DBH* ≥ 4 cm were recorded separately.

##### S1.2.2 Understory vegetation survey

For shrubs below the tree-census threshold (*DBH* < 4 cm), three 3 m × 3 m subplots were established within each plot (Figure S1b). Within each subplot, we recorded species identity and the number of clonal stems. Shrub cover was estimated using the line-intercept protocol of the Fire Effects Monitoring and Inventory System (FIREMON; Lutes et al., 2006). Mean foliage height was calculated from measurements at five randomly selected points within each subplot.

Herbaceous vegetation was characterized using three 1 m × 1 m quadrats established along the diagonal of each plot (Figure S1c), in which species composition, percent cover, and mean height were recorded. To improve representativeness, the selected quadrats encompassed three cover classes: low cover (≤25%), intermediate cover (>25% and ≤50%), and high cover (>50%).

Within each herbaceous quadrat, aboveground biomass was clipped at ground level, and the corresponding root systems were excavated. Aboveground and belowground fractions were cleaned, bagged, oven-dried at 80 °C to constant mass, and weighed separately. These measurements were used to estimate herbaceous biomass density. Shrub biomass was estimated using allometric equations, as described in Section 2.3.4 of the main text.

Naturally regenerated tree saplings with DBH < 4 cm were censused throughout each 20 × 20 m plot, rather than only within the shrub and herbaceous quadrats. Counts were converted to stems ha⁻¹ to quantify regeneration capacity (RC), as described in Section 2.3.7 of the main text.

##### S1.2.3 Soil sampling

Soil sampling was conducted concurrently with the vegetation survey. At the center of each plot, a soil core was collected from the 0–10 cm mineral-soil layer using a corer with a diameter of 5.0 cm to determine maximum water-holding capacity, while humus layer thickness was measured in situ adjacent to the coring point. Samples from the 0–20 cm mineral-soil layer were also collected at five locations within each plot (Figure S1d), composited, and subsampled using the quartering method. After air-drying, removal of roots and stones, grinding, and sieving through a 0.2 mm mesh, the composite samples were analyzed for soil chemical properties. Bulk density and gravel fraction used in soil organic carbon (SOC) stock calculations were determined for the 0–20 cm mineral-soil layer. Thus, the soil component of water conservation (WC) represented the 0–10 cm layer, whereas soil carbon stocks used in carbon storage (CS) and soil fertility maintenance (SF) represented the 0–20 cm layer.

Figure S1. Schematic diagram of plot survey methods: (a) habitat-survey point within the plot; (b) layout of shrub-survey quadrats; (c) herbaceous-layer quadrats; and (d) soil-sampling point

#### S1.3 Acquisition and selection of biotic and abiotic variables

Definitions and measurement methods for candidate variables are provided in Tables S3 and S4. Variable selection is described in Section 2.2 of the main text, and the screening results are presented in Supplementary Section S2.1.

##### S1.3.1 Acquisition of environmental abiotic variables

A total of 12 abiotic variables were considered, comprising six climatic variables and six soil variables (Table S3). Climatic data sources included on-site automatic weather stations (DYNAMET FLOW32A-1K, USA) and ClimateAP (version 3.10). ClimateAP provides location-specific climate estimates through spatial downscaling (Wang et al., 2012; Wang et al., 2017). The ClimateAP-derived variables used here represented means for 1991–2020 and characterized the long-term climatic background of the sampling locations. Soil variables were measured in the laboratory using the methods summarized in Table S3.

Table S3. Description of the climate and soil variables used in the study

| **Category** | **Index** | **Code** | **Unit** | **Functional and ecological significance** | **Method** | **Reference** |
| --- | --- | --- | --- | --- | --- | --- |
| Climate | Mean annual temperature | MAT | ℃ | Represents long-term thermal conditions within a plot; regulates plant growth, decomposition, nutrient cycling, evapotranspiration, and carbon–water coupling, thereby influencing multiple regulating, provisioning, and supporting services. | - | (Banbury Morgan et al., 2021) |
|  | Mean temperature of the warmest month | MWMT | ℃ | Characterizes warm-season thermal conditions relevant to photosynthesis, respiration, and evaporative demand. | - | (Banbury Morgan et al., 2021) |
|  | Mean temperature of the coldest month | MCMT | ℃ | Characterizes cold-season thermal conditions relevant to species establishment, phenology, and overwinter survival. | - | (Banbury Morgan et al., 2021) |
|  | Mean annual precipitation | MAP | mm | Represents long-term water input; regulates soil moisture availability, plant water use, nutrient transport, litter decomposition, and hydrological regulation, thereby supporting water conservation, productivity, carbon sequestration, and soil fertility maintenance. | - | (Fick and Hijmans, 2017) |
|  | Hargreaves reference evapotranspiration | Eref | mm | Estimates atmospheric evaporative demand; reflects potential climatic water loss and plant water demand, thereby mediating the balance between productivity, carbon accumulation, water conservation, and drought-related functional constraints. | - | (Hargreaves and Samani, 1985) |
|  | Hargreaves climatic water deficit | CMD | mm | Quantifies the imbalance between evaporative demand and water supply; indicates moisture limitation and drought stress, which may constrain tree growth, regeneration, soil biological activity, and the simultaneous maintenance of multiple ecosystem services. | - | (Hargreaves and Samani, 1985) |
| Soil | Soil pH | pH | - | Indicates the soil chemical environment; regulates nutrient availability, microbial community composition, enzyme activity, and organic matter decomposition, thereby influencing soil fertility, productivity, carbon cycling, and belowground contributions to EMF | Determined potentiometrically using a pH meter at a soil-to-water ratio of 1:2.5 (w/v) | (Rousk et al., 2010) |
|  | Soil total nitrogen | STN | g·kg^-1^ | Characterizes total soil nitrogen status, providing context for plant nutrition and nutrient cycling; nitrogen availability also depends on its chemical forms and turnover. | Measured using an elemental analyzer (vario MICRO cube, Germany) | (Liu et al., 2014) |
|  | Soil total phosphorus | STP | g·kg^-1^ | Characterizes total soil phosphorus status relevant to plant growth and nutrient cycling; plant availability also depends on phosphorus forms and soil conditions. | Determined by the molybdenum–antimony colorimetric method | (Liu et al., 2014) |
|  | Soil organic carbon | SOC | g·kg^-1^ | Quantifies soil organic carbon concentration, reflecting organic matter status relevant to nutrient retention, soil aggregation, and microbial habitat. | Measured using an elemental analyzer (vario MICRO cube, Germany) | (Liu et al., 2014) |
|  | Soil available phosphorus | SAP | mg·kg^-1^ | Measures Bray-extractable phosphorus as an operational indicator of soil phosphorus availability. | Extracted with hydrochloric acid and ammonium fluoride (Bray method) | (Liu et al., 2014) |
|  | Soil alkaline-hydrolysable nitrogen | SAN | mg·kg^-1^ | Measures alkaline-hydrolysable nitrogen as an index of potential soil nitrogen supply relevant to plant growth and microbial activity. | Determined by the alkaline hydrolysis diffusion method | (Powers, 1980) |

Note: Soil variables were measured in composite samples from the 0–20 cm mineral-soil layer. SOC denotes organic carbon concentration (g kg^-1^), whereas soil fertility maintenance in the multifunctionality assessment was represented by organic carbon stock (t ha^-1^).

##### S1.3.2 Acquisition of stand biotic variables

Twelve biotic variables were considered to characterize tree species diversity and stand structure (Table S4). Spatial indices comprised the uniform angle index, neighborhood comparison index, mingling degree, crowding index, and Hegyi competition index. These indices were calculated using the four-nearest-neighbor tree-unit method, in which each unit comprised a reference tree and its four nearest neighboring trees, irrespective of species identity (Hui et al., 2016). The indices characterize neighborhood geometry, relative tree size, species interspersion, crown overlap, and potential competitive pressure (Hui et al., 2007). Individual-tree values (W_ᵢ_, U_ᵢ_, M_ᵢ_, C_ᵢ_, and CI_ᵢ_) were averaged across reference trees to obtain the corresponding plot-level indices (W_m_, U_m_, M_m_, C_m_, and CI_m_). To reduce edge effects, a 2 m internal buffer was established along each plot boundary. Trees within this buffer were included as neighbors but excluded as reference trees (Dong et al., 2022; Zhou et al., 2009).

Table S4. Description of the diversity and stand variables used in the study

| **Category** | **Index** | **Code** | **Functional and ecological significance** | **Formulation** | **Notes** | **Reference** |
| --- | --- | --- | --- | --- | --- | --- |
| Diversity | Tree species richness | **SR** | Represents taxonomic diversity within a plot; higher SR may enhance niche complementarity, resource-use efficiency, habitat heterogeneity, and functional redundancy, thereby supporting the simultaneous provision of multiple ecosystem services. | $\text{SR}=S$ | *S* is the total number of tree species observed in each plot. | (Gotelli and Colwell, 2001) |
|  | Diameter-based structural diversity | **SDN** | Captures the diversity of stem distribution among diameter classes, reflecting both the number of occupied classes and their relative abundances. |  | *dc* is the number of diameter classes in a plot, *n_j_* is the number of trees in diameter class *j*, and n is the total number of trees in the plot. | (Ali et al., 2016) |
|  | Species–diameter structural diversity | **SDNS** | Captures the joint distribution of stems among species and diameter classes, representing combined compositional and size heterogeneity. |  | *n_sp_* is the number of tree species in the plot, and *n_ij_* is the number of individuals of the *i*-th tree species in the *j*-th diameter class within the plot. | (Staudhammer and LeMay, 2001) |
| Stand | Mean stand age | **Age** | Characterizes variation in stand development and restoration history within the sampled 32–39-year age range. |  | *n_age_* is the number of trees for which age was measured in the plot, and *age_i_* is the age of the *i*-th sampled tree in the plot (years). | (Pregitzer and Euskirchen, 2004) |
|  | Reineke’s stand density index | **SDI** | Characterizes size-adjusted stand stocking and potential competition for growing space. |  | *N* is the total number of trees in each plot, *D_0_* is the reference diameter of 20 cm, and *b* is the self-thinning slope coefficient of 1.605. | (Liu et al., 2022) |
|  | Tree density | **SD** | Directly reflects stand density. | $\text{SD}=\frac{N}{AS}$ | *N* is the total number of trees in the sample plot, and *AS* is the sample plot area, which was 400 m^2^ in this study. | (Reineke, 1933) |
|  | Gini coefficient of *DBH* | **D_Gini_** | Quantifies inequality in tree diameter distribution within a plot; heterogeneous size structures modify canopy light interception, promote vertical resource partitioning, and influence carbon storage, habitat heterogeneity, and multifunctional service provision. |  | *b_ak_* denotes the breast height cross-sectional area of the *k*-th individual in ascending order, *D_Gini_* = 0 when there is no difference between individuals, and *D_Gini_* takes a value infinitely close to 1 when differences are maximized. | (Zhang and Chen, 2015) |
|  | Uniform angle index | **W_i_** | Describes the horizontal spatial distribution pattern of neighboring trees; reflects whether trees are regularly, randomly, or clumpily distributed, thereby influencing local competition, resource capture, regeneration microsites, and spatial heterogeneity in ecosystem functions. |  | *W_i_* is the proportion of four neighborhood angles smaller than the reference angle *α_0_* = 72°. *Z_ij_* = 1 when angle *j* is smaller than α₀ and 0 otherwise. *W_i_* takes values of 0, 0.25, 0.50, 0.75, or 1.00; higher values indicate greater local aggregation. | (Yang et al., 2024) |
|  | Neighborhood comparison index | **U_i_** | Quantifies the relative size dominance of focal trees compared with their nearest neighbors; reflects local competitive hierarchy and asymmetric resource acquisition, thereby affecting growth allocation, stand productivity, regeneration conditions, and service trade-offs. | $\text{U}_{\text{i}}=\frac{1}{4}\sum_{j=1}^{4} K_{ij}$ | *K_ij_* = 1 when neighboring tree *j* is at least as large as focal tree *i* and 0 otherwise. *U_i_* takes values of 0, 0.25, 0.50, 0.75, or 1.00. Higher values indicate a lower relative size position of the focal tree within its neighborhood. | (Zhao et al., 2014) |
|  | Mingling degree | **M_i_** | Measures local interspecific mixing, characterizing neighborhood composition relevant to resource complementarity and competitive interactions. |  | *V_ij_* = 1 when neighboring tree *j* belongs to a different species from focal tree *i* and 0 otherwise. *M_i_* takes values of 0, 0.25, 0.50, 0.75, or 1.00. Higher values indicate a greater proportion of heterospecific neighbors. | (Wang et al., 2021) |
|  | Crowding index | **C_i_** | Quantifies the local crowding or canopy occupancy around individual trees; reflects neighborhood space limitation and crown interference, thereby regulating light availability, growth suppression, understory development, and the balance among productivity, regeneration, and regulating services. |  | *Y_ij_* = 1 when the crown projection of neighboring tree *j* overlaps that of focal tree i and 0 otherwise. *C_i_* takes values of 0, 0.25, 0.50, 0.75, or 1.00. Higher values indicate greater local crown overlap. | (Yu et al., 2024) |
|  | Hegyi competition index | **CI_i_** | Provides a distance-dependent index of neighborhood competitive pressure based on the relative diameters and distances of neighboring trees. |  | *L_ij_* is the distance between focal tree *i* and neighboring tree *j* (m), and *d_i_* and *d_j_* are their respective *DBH*s (cm). The index was calculated using the four nearest neighbors of each focal tree (*n* = 4). | (Liu et al., 2024) |

#### S1.4 Ecological hypotheses, SEM specification, and model refinement

The theoretical structural equation model (SEM) was developed to evaluate direct and indirect associations among restoration pathway, soil conditions, community composition, stand structural attributes, and ecosystem multifunctionality (EMF). The initial conceptual framework was informed by ecological hypotheses and subsequently refined through exploratory model comparisons. Restoration pathway was hypothesized to influence community assembly and stand development through differences in stand origin, regeneration mode, planting history, and management legacy. Accordingly, paths were specified from restoration pathway to species diversity, stand structure, spatial stand structure, structural diversity, and EMF.

Soil nutrient availability can constrain species establishment, tree growth, competitive filtering, and ecosystem service provisioning. Paths linking soil nutrient status to species diversity, stand structure, and EMF were therefore considered ecologically plausible. Species diversity and stand structure were also hypothesized to influence spatial stand structure because neighborhood species mixing depends on both the available species pool and the density-dependent spatial arrangement of individuals. Direct paths from species diversity, stand structure, spatial stand structure, and structural diversity to EMF represented potential associations involving niche complementarity, competition, resource partitioning, and canopy occupation. The ecological rationale for paths retained in the final model is summarized in Table S5.

The model was fitted to all 36 plots as an observed-variable covariance-based SEM using maximum likelihood estimation in the lavaan package in R 4.4.2 (Rosseel, 2012). Soil nutrient status, species diversity, stand structure, spatial stand structure, and structural diversity were represented by soil total phosphorus (STP), tree species richness (SR), stand density index (SDI), mingling degree (Mm), and diameter-based structural diversity (SDN), respectively. EMF-Mean was the terminal response. Each component was represented by a single observed indicator rather than a latent construct. Continuous variables were Z-standardized. Restoration pathway was numerically coded and treated as a continuous predictor in the SEM.

The initial, fully connected implementation of the conceptual framework was saturated, with zero degrees of freedom, and was therefore simplified to obtain a testable model. During preliminary refinement, soil total nitrogen (STN) was excluded because its direct and total associations with EMF were weak. The unsupported restoration pathway → STP path was also removed, and STP was subsequently treated as an exogenous soil variable. These adjustments preceded the candidate-model comparisons summarized in Table S6.

Within the resulting variable set, the model without STP → SDI was designated Model A. Adding this ecologically plausible path produced Model B, improved model fit, and yielded a statistically supported coefficient; the path was therefore retained. The unsupported SR → SDN and SDI → SDN paths were then evaluated for removal, individually and jointly. Removing both paths produced the lowest Akaike information criterion (AIC) and Bayesian information criterion (BIC) values among these candidates without reducing the number of statistically supported paths (Table S6). These modifications followed inspection of model results and were exploratory. Table S5, initially compiled to document the ecological basis of the proposed paths, was subsequently streamlined to correspond to the final model.

Model fit was evaluated using the chi-square statistic (χ^2^), its degrees of freedom and *p*-value, χ^2^/df, the comparative fit index (CFI), the root mean square error of approximation (RMSEA), the standardized root mean square residual (SRMR), and the goodness-of-fit index (GFI). AIC and BIC were used to compare candidate path structures fitted to the same observed variables and dataset. Selection considered model fit, parsimony, and ecological interpretability together; improvement in every fit index was not required.

Standardized path coefficients were reported. Indirect effects were calculated as products of coefficients along the relevant paths and summed where multiple indirect routes connected the same predictor and response. Total effects were calculated as the sum of direct and indirect effects. Direct, indirect, and total effects were evaluated using 5000 non-parametric bootstrap resamples with 95% confidence intervals. These intervals describe uncertainty conditional on the selected model structure. Given the observational design and exploratory model refinement, estimated pathways were interpreted as conditional associations rather than demonstrated causal effects.

Threshold-based sensitivity analyses retained the upstream path structure of the final EMF-Mean model, substituting EMF-T25, EMF-T50, or EMF-T75 as the terminal response. These models were used to examine whether associations with multifunctionality varied across service-performance thresholds.

Table S5. Ecological rationale for paths retained in the final SEM.

| No. | Path retained in the final model | Ecological rationale | References |
| --- | --- | --- | --- |
| 1 | Restoration pathway  →  Species diversity | Differences in species pools, regeneration sources, and management histories may influence community assembly and tree species richness. | (Chesson, 2000; Connell and Slatyer, 1977; Fukami, 2015) |
| 2 | Restoration pathway  →  Stand structure | Planting density, regeneration mode, and thinning history may contribute to differences in stand stocking. | (Connell and Slatyer, 1977; Forrester and Bauhus, 2016; Pretzsch and Schütze, 2021) |
| 3 | Restoration pathway  →  Spatial stand structure | Planting patterns, enrichment planting, and natural recruitment may influence neighborhood species composition. | (Forrester and Bauhus, 2016; Pretzsch, 2022; Pretzsch and Schütze, 2021) |
| 4 | Restoration pathway  →  Structural diversity | Differences in recruitment, growth, and mortality may contribute to variation in diameter-class distributions. | (Forrester and Bauhus, 2016; Ouyang et al., 2023; Wang et al., 2023) |
| 5 | Restoration pathway  → EMF | This path allows for associations with restoration history that are not represented by the measured soil and stand attributes. | (Byrnes et al., 2014; Hooper et al., 2005; Isbell et al., 2011) |
| 6 | Soil nutrient status  →  Species diversity | Soil nutrient conditions may influence species establishment and competitive filtering. | (Chesson, 2000; Fang et al., 2017; Wardle et al., 2004) |
| 7 | Soil nutrient status  →  Stand structure | Soil nutrient conditions may influence tree growth and stand stocking. | (Fang et al., 2017; Pretzsch and Schütze, 2021; Wardle et al., 2004) |
| 8 | Soil nutrient status  → EMF | Nutrient conditions may influence multiple functions through plant growth, carbon accumulation, and belowground processes. | (Fang et al., 2017; Hooper et al., 2005; Wardle et al., 2004) |
| 9 | Species diversity  →  Spatial stand structure | A larger species pool may increase the occurrence of heterospecific neighbors. | (Forrester and Bauhus, 2016; Pretzsch, 2022) |
| 10 | Stand structure  →  Spatial stand structure | Stand stocking may influence inter-tree spacing and neighborhood composition. | (Forrester and Bauhus, 2016; Pretzsch, 2022; Pretzsch and Schütze, 2021) |
| 11 | Species diversity  → EMF | Species differences in resource use and functional contributions may support multiple ecosystem services. | (Jucker et al., 2014; Lefcheck et al., 2015; Loreau and Hector, 2001) |
| 12 | Stand structure  → EMF | Stand stocking may influence resource capture, biomass accumulation, and competition. | (Ouyang et al., 2023; Xu et al., 2024) |
| 13 | Spatial stand structure  → EMF | Neighborhood species mixing may alter local competition and resource complementarity. | (Pretzsch, 2022; Xu et al., 2024) |
| 14 | Structural diversity  → EMF | Diameter-class distributions may reflect differences in stand development, resource capture, and competition relevant to multifunctionality. | (Storch et al., 2018; Xu et al., 2024) |

Note: Arrows indicate hypothesized directions of association rather than demonstrated causal effects. This table presents the ecological rationale for paths retained in the final model. The model-development sequence and subsequent path modifications are documented in Section S1.4 and Table S6.

Table S6. Comparison of candidate SEMs for EMF-Mean

| Model | Path modification | AIC | BIC |
| --- | --- | --- | --- |
| Model A | Model without STP → SDI | 72.279 | 121.368 |
| Model B | Added STP → SDI to Model A | 68.633 | 119.306 |
| Model C | Removed SR → SDN from Model B | 66.794 | 115.883 |
| Model D | Removed SDI → SDN from Model B | 67.064 | 116.153 |
| Final model | Removed both SR → SDN and SDI → SDN from Model B | 65.263 | 112.768 |

Note: All candidate models used the same 36 plots and observed variables. Models C, D, and the final model represent alternative reductions of Model B. Lower Akaike information criterion (AIC) and Bayesian information criterion (BIC) values indicate a better balance between model fit and parsimony. STP, soil total phosphorus; SR, tree species richness; SDI, stand density index; SDN, diameter-based structural diversity.

#### S1.5 Supplementary statistical procedures

Differences in EMF-Mean, EMF-T25, EMF-T50, and EMF-T75 among restoration pathways were assessed separately using one-way analysis of variance followed by Tukey’s post hoc test at *p* < 0.05. Figure 3a presents means ± standard deviations for 12 plots per pathway.

Univariate linear regressions and forward stepwise regressions provided exploratory candidate-variable screening. Stepwise inclusion required a reduction in the small-sample-corrected Akaike information criterion (AICc), *p*-entry < 0.05, and variance inflation factors < 5, with a maximum of four predictors per model. The selected models are reported in Table S8.

For relative-importance analysis, standardized ordinary least-squares (OLS) models were fitted separately for each restoration pathway and EMF metric. Fixed candidate sets comprised STP, M_m_, MAT, SR, and SDN for pure plantations; SDN, SR, Age, pH, and STN for mixed plantations; and SDI, STN, SR, STP, MAT, and M_m_ for secondary forests. The same set was used across all four EMF metrics within each pathway. Lindeman–Merenda–Gold (LMG) contributions were expressed as percentages summing to 100% of the explained variance within each model.

The significance of individual LMG contributions was assessed using 199 permutations. These contribution tests were distinct from coefficient tests in the full candidate OLS models and from the single-predictor linear mixed-effects models (LMMs) used in Figure 4. Relative-importance results informed exploratory ranking and SEM variable selection rather than causal inference.

Pairwise Pearson correlations among the eight ecosystem service (ES) indicators were calculated for all 36 plots and separately for each restoration pathway (*n* = 12). Two-sided *p*-values were adjusted using the Benjamini–Hochberg procedure within each set of 28 service pairs. These exploratory correlations describe covariation rather than imbalance quantified by root mean square deviation (RMSD) and were not adjusted for region. Pooled correlations may reflect both within- and between-pathway variation. All eight indicators retained equal weight in EMF.

### S2 Supplementary Results

#### S2.1 Variable-screening results

Variable screening considered pairwise correlations, variance inflation factors (VIFs), and ecological relevance, informed by previous studies (Costa et al., 2026; Lan et al., 2023; Sanaei et al., 2021; Wu et al., 2025). Ten variables were ultimately retained: mean annual temperature (MAT), soil pH (pH), soil total nitrogen (STN), soil total phosphorus (STP), mean stand age (Age), stand density index (SDI), mingling degree (M_m_), Hegyi competition index (CI_m_), tree species richness (SR), and diameter-based structural diversity (SDN). Absolute pairwise correlation coefficients among the retained variables were below 0.70 (Figure S2). VIF values ranged from 1.59 to 3.41 (Table S7), satisfying the specified screening criterion of VIF < 3.5.

Figure S2. Pearson correlations among retained candidate explanatory variables

Note: Lower-triangle values are Pearson’s correlation coefficients; colors range from red (lower values) to blue (higher values). Upper-triangle asterisks indicate significance: **p* < 0.05; ***p* < 0.01. Variable abbreviations are defined in Table S7.

Table S7. Descriptive statistics and variance inflation factors of retained candidate explanatory variables

| **Category** | **Index** | **Code** | **Unit** | **Mean** | **SD** | **VIF** |
| --- | --- | --- | --- | --- | --- | --- |
| Climate | Mean annual temperature | MAT | ℃ | 17.78 | 2.82 | 2.54 |
| Soil | Soil pH | pH | - | 4.71 | 0.64 | 3.41 |
|  | Soil total nitrogen | STN | g·kg^-1^ | 0.27 | 0.11 | 1.60 |
|  | Soil total phosphorus | STP | g·kg^-1^ | 0.99 | 0.48 | 2.38 |
| Diversity | Tree species richness | SR | species·plot^-1^ | 11.67 | 6.71 | 2.75 |
|  | Diameter-based structural diversity | SDN | - | 1.89 | 0.15 | 2.27 |
| Stand | Stand age | Age | Year | 35.00 | 2.55 | 2.49 |
|  | Reineke’s stand density index | SDI | stems·ha^-1^ | 1463.34 | 324.08 | 2.26 |
|  | Mingling degree | M_m_ | - | 0.50 | 0.03 | 1.59 |
|  | Hegyi competition index | CI_m_ | - | 0.59 | 0.21 | 2.53 |

Note: SD, standard deviation; VIF, variance inflation factor. Variable definitions are provided in Tables S3 and S4.

#### S2.2 Linear regression results: univariate and forward stepwise regressions

Univariate linear regression was first used as an exploratory screening step to identify candidate variables associated with ecosystem multifunctionality (EMF) within each restoration pathway. Models were fitted separately for pure plantations, mixed plantations and secondary forests, using EMF-Mean, EMF-T25, EMF-T50 and EMF-T75 as response variables. Only significant univariate associations (*p* < 0.05) are shown in Figure S3. Forward stepwise regression identified variable combinations associated with each EMF metric. Retained coefficient estimates are shown in Figure S4, and selected predictors and model summaries are reported in Table S8.

In pure plantations, soil total phosphorus and mingling degree were the most frequently retained variables. Soil total phosphorus was retained in the EMF-Mean, EMF-T25 and EMF-T75 models, whereas mingling degree was retained in the EMF-Mean and EMF-T50 models. Tree species richness, diameter-based structural diversity and mean annual temperature were retained only for specific EMF metrics. The retained coefficients for soil total phosphorus and mingling degree were positive.

In mixed plantations, the EMF-Mean model retained diameter-based structural diversity and soil pH, both with negative coefficients. No predictor was retained for EMF-T25. The EMF-T50 model retained stand age and tree species richness with negative coefficients and soil total nitrogen with a positive coefficient. Tree species richness and diameter-based structural diversity were retained in the EMF-T75 model, both with negative coefficients.

In secondary forests, stand density index and soil total nitrogen were consistently retained in the EMF-Mean and EMF-T50 models, and both showed negative associations with EMF. The EMF-T25 model retained tree species richness, soil total phosphorus, and mean annual temperature, whereas the EMF-T75 model retained mingling degree alone, with a positive coefficient.

These regressions provided an exploratory basis for candidate selection. Region-adjusted single-predictor LMM associations and LMG contributions from fixed-candidate multiple OLS models are presented in Figures 4 and 5, respectively. SEM specification and subsequent modifications are documented in Section S1.4.

Figure S3. Significant univariate associations between candidate variables and ecosystem multifunctionality across restoration pathways

Note: (a) pure plantations, (b) mixed plantations, and (c) secondary forests. Points and horizontal bars indicate standardized regression coefficients and 95% confidence intervals; only associations with *p* < 0.05 are shown. Yellow/orange, green, and blue identify the three restoration pathways, respectively. Mean, T25, T50, and T75 denote EMF-Mean and multifunctionality at the 25%, 50%, and 75% thresholds. Variable abbreviations are defined in Table S7.

Figure S4. Candidate correlates of EMF retained by forward stepwise regression across restoration pathways

Note: (a) pure plantations, (b) mixed plantations, and (c) secondary forests. Points and horizontal bars indicate coefficients and 95% confidence intervals; vertical dashed lines mark zero. Colors and EMF labels follow Figure S3. No predictor was retained for EMF-T25 in mixed plantations. Model summaries are provided in Table S8.

Table S8. Summary of final forward stepwise regression models

| **Restoration pathway** | **EMF metric** | **Selected variables** | ***R^2^*** | **Adjusted *R^2^*** | **AICc** | **BIC** |
| --- | --- | --- | --- | --- | --- | --- |
| Pure plantations | EMF-Mean | STP + M_m_ + MAT | 0.832 | 0.769 | -8.761 | -12.536 |
|  | EMF-T25 | STP + SR | 0.596 | 0.507 | -2.932 | -4.478 |
|  | EMF-T50 | M_m_ + SDN | 0.651 | 0.573 | -4.672 | -6.217 |
|  | EMF-T75 | STP + SDN | 0.613 | 0.527 | -3.425 | -4.97 |
| Mixed plantations | EMF-Mean | SDN + pH | 0.736 | 0.678 | -8.034 | -9.58 |
|  | EMF-T25 | None | 0 | 0 | 1.356 | 1.441 |
|  | EMF-T50 | Age + SR + STN | 0.811 | 0.741 | -7.353 | -11.127 |
|  | EMF-T75 | SR + SDN | 0.802 | 0.758 | -11.476 | -13.021 |
| Secondary forests | EMF-Mean | SDI + STN | 0.858 | 0.826 | -15.457 | -17.003 |
|  | EMF-T25 | SR + STP + MAT | 0.791 | 0.713 | -6.131 | -9.906 |
|  | EMF-T50 | STN + SDI | 0.652 | 0.575 | -4.716 | -6.261 |
|  | EMF-T75 | M_m_ | 0.396 | 0.335 | -1.756 | -2.12 |

Note: Models were fitted separately for each restoration pathway and EMF metric. Selection criteria were *p*-entry < 0.05, improvement in the small-sample corrected Akaike information criterion (AICc), VIF < 5, and a maximum of four predictors. BIC, Bayesian information criterion. “None” indicates that no predictor met the selection criteria and the intercept-only model was retained.

#### S2.3 Threshold-based SEM sensitivity analysis

Threshold-based sensitivity analyses retained the upstream structure of the EMF-Mean model and replaced the terminal response with EMF-T25, EMF-T50, or EMF-T75 (Figure S5). These models assessed whether EMF-related associations persisted across thresholds.

The EMF-T25 model explained 20.5% of the variation in multifunctionality (*R^2^* = 0.205), and no direct path to EMF-T25 reached statistical significance. The EMF-T50 and EMF-T75 models explained 67.2% and 54.7% of the variation, respectively. Mingling degree was positively associated with EMF-T50 (β = 0.584, *p* = 0.001) and EMF-T75 (β = 0.514, *p* = 0.010), whereas diameter-based structural diversity was negatively associated with EMF-T50 (β = −0.323, *p* = 0.012) and EMF-T75 (β = −0.454, *p* = 0.003).

The positive associations with mingling degree and negative associations with diameter-based structural diversity at the 50% and 75% thresholds were consistent in direction with those in the EMF-Mean model. Differences in coefficient magnitudes across thresholds are descriptive and should not be interpreted as statistically tested differences in association strength.

Figure S5. Threshold-based structural equation models and standardized direct and indirect associations

Note: Panels (a, b), (c, d), and (e, f) correspond to EMF-T25, EMF-T50, and EMF-T75, respectively. Red and blue solid arrows indicate significant positive and negative paths; gray dashed arrows indicate nonsignificant paths. Numbers are standardized coefficients; **p* < 0.05 and ***p* < 0.01. Solid and hatched bars represent standardized direct and summed indirect estimates, respectively. *R^2^* indicates explained variance. GFI, goodness-of-fit index; χ^2^/df, chi-square divided by degrees of freedom. Variable abbreviations are defined in Table S7.

#### S2.4 Covariation among ecosystem-service indicators

Across all 36 plots, CS and SP were strongly positively correlated (r = 0.937, Benjamini–Hochberg-adjusted *q* < 0.001), and this was the only pooled association remaining significant after correction (Figure S6). Within pathways, significant positive correlations occurred between CR and H′ and between CS and SP in pure plantations. CR and H′ were negatively correlated in mixed plantations. In secondary forests, SC and CR were negatively correlated, whereas CS–SP and RC–SF were positively correlated. These results describe service covariation and complement the RMSD assessment of differences in relative service levels; nonsignificant correlations do not establish independence.

Figure S6. Pairwise correlations among ecosystem-service indicators

Note: Pearson correlations for (a) all plots and (b–d) pure plantations, mixed plantations, and secondary forests. Red and blue indicate positive and negative correlations. Asterisks denote Benjamini–Hochberg-adjusted significance within each set of 28 pairs (**q* < 0.05; ***q* < 0.01). Service abbreviations follow Table 1.

### S3 References

Ali, A., Yan, E. R., Chen, H. Y. H., Chang, S. X., Zhao, Y. T., Yang, X. D., et al. (2016). Stand structural diversity rather than species diversity enhances aboveground carbon storage in secondary subtropical forests in eastern China. Biogeosciences 13, 4627–4635. doi: 10.5194/bg-13-4627-2016.

Banbury Morgan, R., Herrmann, V., Kunert, N., Bond-Lamberty, B., Muller-Landau, H. C., and Anderson-Teixeira, K. J. (2021). Global patterns of forest autotrophic carbon fluxes. Glob. Change Biol. 27, 2840–2855. doi: 10.1111/gcb.15574.

Byrnes, J. E. K., Gamfeldt, L., Isbell, F., Lefcheck, J. S., Griffin, J. N., Hector, A., et al. (2014). Investigating the relationship between biodiversity and ecosystem multifunctionality: challenges and solutions. Methods Ecol. Evol. 5, 111–124. doi: 10.1111/2041-210X.12143.

Chesson, P. (2000). Mechanisms of maintenance of species diversity. Annu. Rev. Ecol. Syst. 31, 343–366. doi: 10.1146/annurev.ecolsys.31.1.343.

Connell, J. H., and Slatyer, R. O. (1977). Mechanisms of succession in natural communities and their role in community stability and organization. Am. Nat. 111, 1119–1144. doi: 10.1086/283241.

Costa, J. R. S., Brancalion, P. H. S., Joly, F.-X., Simões, L. H. P., Bonfanti, J., le Maire, G., et al. (2026). Forest ecosystem multifunctionality: a systematic review of measures and drivers. Curr. For. Rep. 12, 2. doi: 10.1007/s40725-025-00266-4.

Dong, L., Bettinger, P., and Liu, Z. (2022). Optimizing neighborhood-based stand spatial structure: four cases of boreal forests. For. Ecol. Manage. 506, 119965. doi: 10.1016/j.foreco.2021.119965.

Fang, X.-M., Zhang, X.-L., Zong, Y.-Y., Zhang, Y., Wan, S.-Z., Bu, W.-S., et al. (2017). Soil phosphorus functional fractions and tree tissue nutrient concentrations influenced by stand density in subtropical Chinese fir plantation forests. PLoS One 12, e0186905. doi: 10.1371/journal.pone.0186905.

Fick, S. E., and Hijmans, R. J. (2017). WorldClim 2: new 1-km spatial resolution climate surfaces for global land areas. Int. J. Climatol. 37, 4302–4315. doi: 10.1002/joc.5086.

Forrester, D. I., and Bauhus, J. (2016). A review of processes behind diversity–productivity relationships in forests. Curr. For. Rep. 2, 45–61. doi: 10.1007/s40725-016-0031-2.

Fukami, T. (2015). Historical contingency in community assembly: integrating niches, species pools, and priority effects. Annu. Rev. Ecol. Evol. Syst. 46, 1–23. doi: 10.1146/annurev-ecolsys-110411-160340.

Gotelli, N. J., and Colwell, R. K. (2001). Quantifying biodiversity: procedures and pitfalls in the measurement and comparison of species richness. Ecol. Lett. 4, 379–391. doi: 10.1046/j.1461-0248.2001.00230.x.

Hargreaves, G. H., and Samani, Z. A. (1985). Reference crop evapotranspiration from temperature. Appl. Eng. Agric. 1, 96–99. doi: 10.13031/2013.26773.

Hooper, D. U., Chapin, F. S., III, Ewel, J. J., Hector, A., Inchausti, P., Lavorel, S., et al. (2005). Effects of biodiversity on ecosystem functioning: a consensus of current knowledge. Ecol. Monogr. 75, 3–35. doi: 10.1890/04-0922.

Hui, G. Y., Li, L., Zhao, Z. H., and Dang, P. X. (2007). The comparison of methods in analysis of the tree spatial distribution pattern. Acta Ecol. Sin. 27, 4717–4728.

Hui, G. Y., von Gadow, K., Zhao, Z. H., Hu, Y. B., Xu, H., Li, Y. F., et al. (2016). Principles of Structure-Based Forest Management. Beijing: China Forestry Publishing House.

Isbell, F., Calcagno, V., Hector, A., Connolly, J., Harpole, W. S., Reich, P. B., et al. (2011). High plant diversity is needed to maintain ecosystem services. Nature 477, 199–202. doi: 10.1038/nature10282.

Jucker, T., Bouriaud, O., Avacaritei, D., and Coomes, D. A. (2014). Stabilizing effects of diversity on aboveground wood production in forest ecosystems: linking patterns and processes. Ecol. Lett. 17, 1560–1569. doi: 10.1111/ele.12382.

Lan, J., Lei, X., He, X., Gao, W., and Guo, H. (2023). Multiple mechanisms drive biodiversity-ecosystem service multifunctionality but the dominant one depends on the level of multifunctionality for natural forests in Northeast China. For. Ecol. Manage. 542, 121101. doi: 10.1016/j.foreco.2023.121101.

Lefcheck, J. S., Byrnes, J. E. K., Isbell, F., Gamfeldt, L., Griffin, J. N., Eisenhauer, N., et al. (2015). Biodiversity enhances ecosystem multifunctionality across trophic levels and habitats. Nat. Commun. 6, 6936. doi: 10.1038/ncomms7936.

Liu, D., Zhou, C. F., He, X., Zhang, X. H., Feng, L. Y., and Zhang, H. R. (2022). The effect of stand density, biodiversity, and spatial structure on stand basal area increment in natural spruce-fir-broadleaf mixed forests. Forests 13, 162. doi: 10.3390/f13020162.

Liu, X., Meng, W., Liang, G., Li, K., Xu, W., Huang, L., et al. (2014). Available phosphorus in forest soil increases with soil nitrogen but not total phosphorus: evidence from subtropical forests and a pot experiment. PLoS One 9, e88070. doi: 10.1371/journal.pone.0088070.

Liu, Y., Zeng, Y., Wang, P., He, J., Li, P., and Liang, Y. (2024). The spatial pattern of Populus euphratica competition based on competitive exclusion theory. Front. Plant Sci. 15:1276489. doi: 10.3389/fpls.2024.1276489.

Loreau, M., and Hector, A. (2001). Partitioning selection and complementarity in biodiversity experiments. Nature 412, 72–76. doi: 10.1038/35083573.

Lutes, D. C., Keane, R. E., Caratti, J. F., Key, C. H., Benson, N. C., Sutherland, S., et al. (2006). FIREMON: Fire Effects Monitoring and Inventory System. General Technical Report RMRS-GTR-164-CD. Fort Collins, CO: U.S. Department of Agriculture, Forest Service, Rocky Mountain Research Station. doi: 10.2737/RMRS-GTR-164.

Ouyang, S., Gou, M., Lei, P., Liu, Y., Chen, L., Deng, X., et al. (2023). Plant functional trait diversity and structural diversity co-underpin ecosystem multifunctionality in subtropical forests. For. Ecosyst. 10, 100093. doi: 10.1016/j.fecs.2023.100093.

Powers, R. F. (1980). Mineralizable soil nitrogen as an index of nitrogen availability to forest trees. Soil Sci. Soc. Am. J. 44, 1314–1320. doi: 10.2136/sssaj1980.03615995004400060037x.

Pregitzer, K. S., and Euskirchen, E. S. (2004). Carbon cycling and storage in world forests: biome patterns related to forest age. Glob. Change Biol. 10, 2052–2077. doi: 10.1111/j.1365-2486.2004.00866.x.

Pretzsch, H. (2022). Mixing degree, stand density, and water supply can increase the overyielding of mixed versus monospecific stands in Central Europe. For. Ecol. Manage. 503, 119741. doi: 10.1016/j.foreco.2021.119741.

Pretzsch, H., and Schütze, G. (2021). Tree species mixing can increase stand productivity, density and growth efficiency and attenuate the trade-off between density and growth throughout the whole rotation. Ann. Bot. 128, 767–786. doi: 10.1093/aob/mcab077.

Reineke, L. H. (1933). Perfecting a stand-density index for even-aged forests. J. Agric. Res. 46, 627–638.

Rosseel, Y. (2012). lavaan: an R package for structural equation modeling. J. Stat. Softw. 48, 1–36. doi: 10.18637/jss.v048.i02.

Rousk, J., Bååth, E., Brookes, P. C., Lauber, C. L., Lozupone, C., Caporaso, J. G., et al. (2010). Soil bacterial and fungal communities across a pH gradient in an arable soil. ISME J. 4, 1340–1351. doi: 10.1038/ismej.2010.58.

Sanaei, A., Ali, A., Yuan, Z., Liu, S., Lin, F., Fang, S., et al. (2021). Context-dependency of tree species diversity, trait composition and stand structural attributes regulate temperate forest multifunctionality. Sci. Total Environ. 757, 143724. doi: 10.1016/j.scitotenv.2020.143724.

Staudhammer, C. L., and LeMay, V. M. (2001). Introduction and evaluation of possible indices of stand structural diversity. Can. J. For. Res. 31, 1105–1115. doi: 10.1139/x01-033.

Storch, F., Dormann, C. F., and Bauhus, J. (2018). Quantifying forest structural diversity based on large-scale inventory data: a new approach to support biodiversity monitoring. For. Ecosyst. 5, 34. doi: 10.1186/s40663-018-0151-1.

Wang, H.-X., Huang, S.-X., Zhang, S.-S., Peng, H., and Cao, K.-F. (2021). Localized neighborhood species mingling is correlated with individual tree size inequality in natural forests in South China. Ann. For. Sci. 78, 102. doi: 10.1007/s13595-021-01111-x.

Wang, J., Liu, H., Yang, Q., Shen, G., Zhu, X., Xu, Y., et al. (2023). Topography and structural diversity regulate ecosystem multifunctionality in a subtropical evergreen broadleaved forest. Front. For. Glob. Change 6:1309660. doi: 10.3389/ffgc.2023.1309660.

Wang, T., Hamann, A., Spittlehouse, D. L., and Murdock, T. Q. (2012). ClimateWNA—high-resolution spatial climate data for western North America. J. Appl. Meteorol. Climatol. 51, 16–29. doi: 10.1175/JAMC-D-11-043.1.

Wang, T., Wang, G., Innes, J. L., Seely, B., and Chen, B. (2017). ClimateAP: an application for dynamic local downscaling of historical and future climate data in Asia Pacific. Front. Agric. Sci. Eng. 4, 448–458. doi: 10.15302/J-FASE-2017172.

Wardle, D. A., Bardgett, R. D., Klironomos, J. N., Setälä, H., van der Putten, W. H., and Wall, D. H. (2004). Ecological linkages between aboveground and belowground biota. Science 304, 1629–1633. doi: 10.1126/science.1094875.

Wu, J., Xiang, Q., Xia, Y., Lei, J., Ai, X., Wang, Z., et al. (2025). Effects of stand structural changes on vegetation carbon sequestration capacity during secondary succession in subtropical evergreen–deciduous broadleaved mixed forests in central China. J. Environ. Manage. 396, 128016. doi: 10.1016/j.jenvman.2025.128016.

Xu, Y., Chen, H. Y. H., Qiao, X., Zhang, Y., and Jiang, M. (2024). The control of external and internal canopy structural heterogeneity on diversity and productivity relationship in a subtropical forest. For. Ecosyst. 11, 100246. doi: 10.1016/j.fecs.2024.100246.

Yang, B., Ma, R., Zhai, J., Du, J., Bai, J., and Zhang, W. (2024). Stand spatial structure is more important than species diversity in enhancing the carbon sink of fragile natural secondary forest. Ecol. Indic. 158, 111449. doi: 10.1016/j.ecolind.2023.111449.

Yu, J., Xu, L., Shu, Q., Luo, S., and Xi, L. (2024). Spatial effects analysis of natural forest canopy cover based on spaceborne LiDAR and geostatistics. Front. Plant Sci. 15:1361297. doi: 10.3389/fpls.2024.1361297.

Zhang, Y., and Chen, H. Y. H. (2015). Individual size inequality links forest diversity and above-ground biomass. J. Ecol. 103, 1245–1252. doi: 10.1111/1365-2745.12425.

Zhao, Z., Hui, G., Hu, Y., Li, Y., and Wang, H. (2014). Method and application of stand spatial advantage degree based on the neighborhood comparison. J. Beijing For. Univ. 36, 78–82. doi: 10.13332/j.1000-1522.2014.01.020.

Zhou, H. M., Hui, G. Y., Zhao, Z. H., and Hu, Y. B. (2009). Treatment methods of plot boundary trees in spatial forest structure analysis. Sci. Silvae Sin. 45, 1–5. doi: 10.11707/j.1001-7488.200900201.
